# Electrospun PVP/HPBCD nanofiber topical drug delivery platform for enhanced skin permeability and anti-pollution bioactivity of *Artocarpus altilis* extract

**DOI:** 10.1080/10717544.2025.2610654

**Published:** 2026-01-06

**Authors:** Chun-Yin Yang, Chih-Hua Tseng, Feng-Lin Yen

**Affiliations:** a Department of Pharmacy and Master Program, Collage of Pharmacy and Health Care, Tajen University, Pingtung County, Taiwan; b School of Pharmacy, College of Pharmacy, Kaohsiung Medical University, Kaohsiung, Taiwan; c Department of Medical Research, Kaohsiung Medical University Hospital, Kaohsiung City, Taiwan; d Department of Fragrance and Cosmetic Science, College of Pharmacy, Kaohsiung Medical University, Kaohsiung, Taiwan; e Institute of Biomedical Sciences, National Sun Yat-Sen University, Kaohsiung City, Taiwan; f College of Professional Studies, National Pingtung University of Science and Technology, Pingtung County, Taiwan

**Keywords:** *Artocarpus altilis*, electrospun nanofibers, skin permeation enhancement, topical drug delivery, particulate matter

## Abstract

*Artocarpus altilis* methanolic extract (AAM) exhibits potent protective effects against particulate matter (PM)-induced skin damage; however, its poor aqueous solubility and limited skin permeability restrict its topical bioavailability. To overcome these limitations, we developed a polymer-based drug delivery system by fabricating electrospun nanofibers composed of polyvinylpyrrolidone (PVP), hydroxypropyl-*β*-cyclodextrin (HPBCD), and AAM. The optimized formulation engineering strategy enhanced AAM solubility via increased surface area, reduced crystallinity, and hydrogen bonding interactions with HPBCD/PVP. The nanofiber matrix also provided an occlusive effect, improving skin hydration and facilitating transdermal diffusion through the stratum corneum. In vitro studies demonstrated improved cellular uptake, greater permeability, and enhanced antioxidant activity, leading to superior anti-pollution efficacy compared to raw AAM in a PM-induced HaCaT keratinocyte model. These results highlight AAM-loaded electrospun nanofibers (ANFs) as a biodegradable, and environmentally sustainable platform for delivering plant-derived bioactive ingredient, offering high potential for advanced topical formulations targeting pollution-induced skin aging.

## Introduction

The increasing demand for natural-based cosmeceuticals and pharmaceuticals has drawn significant attention to the diverse bioactivities of plant extracts. *Artocarpus altilis* methanolic extract (AAM), for example, has been extensively studied for its remarkable photoprotective, anticancer, and skin-whitening effects and possess anti-pollution activities (Fu et al. 2014; Lee et al. 2013; Tzeng et al. 2015; Yang et al. 2022). However, AAM present major challenges in topical formulations, as limited solubility and crystallisation can reduce bioavailability, hinder skin penetration, and compromise formulation stability, which collectively restrict the practical application of AAM in advanced topical products.

Conventional strategies to enhance skin penetration include chemical penetration enhancers, such as alcohols, glycols, surfactants, or terpenes, which disrupt highly organised lipid structure of the stratum corneum to improve absorption (Lane 2013). While effective, these agents frequently induce skin irritation or inflammatory responses, and potential long-term damage to the skin barrier. Similarly, mechanical approaches, such as microneedles, abrasion, or laser radiation, though capable of bypassing the stratum corneum, create micro-channels that compromise the skin's natural barrier function. This can result in transepidermal water loss (TEWL), reduced hydration, and increased vulnerability to environmental stressors (Brown et al. 2006). Therefore, there is a growing need for safe, efficient, and biocompatible topical delivery systems that maximise absorption of poorly soluble actives without impairing barrier integrity.

Electrospun nanofibers offer a versatile strategy to overcome these challenges, providing high surface-area-to-volume ratio, tunable porosity, and controlled release properties. By incorporating active pharmaceutical ingredients (APIs) into a nanofiber matrix, Electrospin effectively enhances the dissolution rate, improves solubility, and promotes sustained release, thereby increasing bioavailability and skin permeation (Ibrahim and Klingner 2020). Selection of polymeric materials and optimisation of electrospinning parameters are critical to achieving desirable nanofiber properties, such as morphology, mechanical strength, and API encapsulation efficiency. Cyclodextrins (CDs), are cyclic oligosaccharides with a unique toroidal structure, featuring hydrophobic inner cavities and hydrophilic exteriors, are widely employed to enhance solubility and bioavailability while reducing toxicity and improving stability (Carrier et al. 2007; Loftsson et al. 2005). Polyvinylpyrrolidone (PVP), a non-toxic amphipathic polymer that not only provides the necessary viscosity for the electrospinning process but also functions as an effective amorphous solid dispersion excipient. PVP's ability to inhibit crystallisation and maintain the API in a high-energy, amorphous state is crucial for enhancing solubility (Zoghbi et al. 2017). The combination of hydroxypropyl-*β*-cyclodextrin (HPBCD) and PVP has been shown to synergistically improve solubilisation efficiency compared to HPBCD alone (Mura et al. 2001).

In this study, we aimed to develop AAM-loaded electrospun nanofibers (ANFs) as a topical delivery system to enhance water solubility, skin permeability, and anti-pollution bioactivity. We characterised the surface morphology and physicochemical properties of ANFs and evaluated their skin penetration. Furthermore, we assessed the antioxidant and anti-inflammatory activities of ANFs in a particulate matter (PM)-induced human keratinocyte model, providing insights into their potential as advanced topical formulations.

## Materials and methods

### Chemicals and reagents

Artocarpin with a purity greater than 95% was provided by Prof. Chih-Hua Tseng (School of Pharmacy, College of Pharmacy, Kaohsiung Medical University, Kaohsiung, Taiwan). Polyvinylpyrrolidone (PVP) with an average molecular weight (MW) of approximately 1,300,000 was purchased from Sigma-Aldrich (St. Louis, MO, USA). Hydroxypropyl-*β*-cyclodextrin (HPBCD) was obtained from Zibo Qianhui Biological Technology Co. Ltd. (Shandong, China). Particulate matter (PM; SRM 1649b) was purchased from the National Institute of Standards and Technology (Gaithersburg, MD, USA). Dimethyl sulfoxide (DMSO) and methanol were purchased from Aencore Chemicals (Surrey Hills, Australia). All other chemicals and reagents were of analytical grade.

### Preparation of Artocarpus altilis Methanolic Extract (AAM)

The dry and chipped heartwood of *A. altilis* was provided by the Tainan District Agricultural Research and Extension Station, Tainan City, Taiwan. The heartwood was extracted with methanol at a solid-to-solvent ratio of 1:20 *(w/v)*. The mixture was sonicated for 1 hour, and the extract was subsequently filtered through a Whatman Grade 4 filter paper. The residue was re-extracted with a fresh portion of methanol. The pooled methanolic extracts were concentrated using a rotary vacuum evaporator and then freeze-dried to obtain the AAM powder. The extraction results are shown in Supplementary Data (Figure S1).

### Fabrication of AAM-Loaded Electrospun Nanofibers (ANFs)

AAM-loaded electrospun nanofibers (ANFs) were prepared using a blend of PVP, HPBCD, and AAM. First, 50 mg of AAM and 1 g of HPBCD were dissolved in 10 mL of ethanol with continuous stirring for 1 hour. Subsequently, varying ratios of PVP were added and stirred until a homogeneous polymer solution was obtained. This mixed solution was then transferred to a plastic syringe and placed on a syringe pump.

Electrospinning was performed using FES-COS equipment (Falco Tech Enterprise Co., Taipei, Taiwan). The process utilised a metallic plate collector covered with aluminium foil. Electrospinning was conducted at 25 ± 2 °C and 45%–55% relative humidity. The electrospinning parameters for different formulations are summarised in Table 1. A higher polymer ratio increases the viscosity of the polymer solution, requiring a higher applied voltage to drive the electrospinning process. The resulting nanofiber mats were collected and stored in moisture-proof containers at 24 ± 2 °C to prevent degradation.

**Table 1.** The composition and electrospinning condition of different ratio ANFs.

	Ratio (AAM:PVP:HPBCD, *w/w/w*)	Voltage (kV)	Flow rate (mL/min)	Needle-to-collector distance (cm)
ANF-2%	1:4:20	13	0.025	10
ANF-4%	1:8:20	13	0.033	10
ANF-6%	1:12:20	13	0.04	10

### High-performance liquid chromatography (HPLC) analysis

The HPLC analysis system (LaChrom Elite L-2000, Hitachi) was equipped with an injection pump (L-2130, Hitachi), autosampler (L-2200, Hitachi), UV–visible detector (L-2420, Hitachi), and Mightysil RP-18 GP column (5 µm particle diameter, 4.6 mm i.d. × 250 mm). The mobile phase contained methanol and deionized distilled water at a ratio of 9:1 *(v/v)*, and the analysis was performed in isocratic mode at a flow rate of 1 mL/min. Artocarpin was detected at a wavelength of 288 nm. A standard curve for artocarpin and AAM was established in the concentration range of 0.05−100 µg/mL using methanol as the solvent.

### Water solubility and yield

For water solubility determination, 1 mg of raw AAM or an equivalent amount of ANFs were dissolved in 1 mL of deionized water. Each solution was shaken for 30 minutes, filtered through a 0.45-µm PVDF membrane, and then diluted tenfold for HPLC analysis.

To determine the yield, different ANF formulations (containing an equivalent of 1 mg of AAM) were dissolved in 1 mL of methanol and diluted 10-fold for HPLC quantification. The yield was calculated using the following equation:

Yield(%)=(C_AAM×V_ANF)/W_ANF×100

where C_AAM_ is the detected concentration of AAM in ANF, V_ANF_ is the volume of ANF solution, and W_ANF_ is the theoretical content of AAM in ANF.

### Encapsulation efficiency

For encapsulation efficiency, ANFs (containing an equivalent of 1 mg of AAM) were dissolved in deionized water and centrifuged at 10 000 rpm for 10 min (Centrifuge 5430 R, Eppendorf, Hamburg, Germany) using a Nanosep centrifugal filter device with a 10 K Omega membrane. The unencapsulated AAM in the filtrate was quantified by HPLC. The encapsulation efficiency was calculated using the following equation:

Encapsulationefficiency(%)=⟦W_TANF−W⟧_FAAM/W_TANF×100

where W_TANF_ is the theoretical content of AAM in ANF and W_FAAM_ represents the detected concentration of free AAM.

### Morphological characterisation

The surface morphologies of raw AAM, HPBCD, PVP, and ANFs were observed using a scanning electron microscope (SEM; Hitachi S-4700, Tokyo, Japan). Samples were mounted on a carbon-conductive tape and sputter-coated with an Au/Pd layer prior to examination. The average fibre diameter was calculated by measuring 30 random fibres using ImageJ software (National Institutes of Health, Bethesda, MD, USA).

The particle morphology of ANFs dissolved in water was visualised with a transmission electron microscope (TEM; JEM-2000EXII; JEOL Co., Tokyo, Japan). A 200 µL sample of a 100 µg/mL ANF aqueous solution (diluted fivefold) was dropped onto a 200-mesh carbon-coated copper grid. To enhance the contrast of the nanostructures, the dried grid was negatively stained. The samples were covered with 0.5% (w/v) phosphotungstic acid solution onto the sample side of the grids. The excess stain was wicked away, and grids were dried at 26 °C for 1 hour before imaging.

### Crystalline state and molecular interactions

To confirm the successful conversion of crystalline AAM to an amorphous form within the nanofiber matrix, X-ray diffraction (XRD) analysis was performed. The crystallinity changes of AAM, HPBCD, PVP, physical mixtures, and ANFs was analysed using an X-ray diffractometer (Siemens D5000, Siemens, Munich, Germany). The XRD patterns were recorded using nickel-filtered Cu Kα radiation at a 2θ range of 5° to 50°. The voltage and current were set at 40 kV and 40 mA, respectively.

Characteristic peak shifts or disappearance were identified as evidence of potential hydrogen bonding or other intermolecular interactions between AAM, HPBCD, and PVP, crucial for the stability of the amorphous dispersion. Molecular interactions were investigated via Fourier-transform infrared spectroscopy (FT-IR) spectroscopy using an ALPHA II FT-IR Spectrometer (Bruker, Billerica, MA, USA). To assess potential hydrogen-bonding interactions between AAM and the polymer matrix, samples were prepared as KBr pellets and analysed by FT-IR spectroscopy. The spectra were scanned from 500−4,000 cm^−1^ to identify changes in functional groups.

### Antioxidant activity (DPPH free radical scavenging assay)

The antioxidant activity of AAM was evaluated using the 2,2-diphenyl-1-picrylhydrazyl (DPPH) free radical scavenging assay. Sample solutions (100 µL) were mixed with 100 µL of a 100 µM DPPH solution in ethanol in a 96-well plate and incubated for 30 minutes in the dark. Absorbance was measured at 517 nm, and the DPPH scavenging activity was calculated using the following equation:

$$\mathrm{DPPH}\;\mathrm{scavenging}\;\mathrm{activity}\;\left(\%\right)=\frac{OD_{\mathrm{control}}-OD_{\mathrm{sample}}}{OD_{\mathrm{control}}}\times 100\%$$

where OD stands for optical density.

### *Ex vivo* skin penetration study

Skin penetration of AAM was determined using Franz diffusion cells as described previously (Yang et al. 2021). The methodology was modified from the guidelines for percutaneous absorption/penetration from European Cosmetic and Perfumery Association.

### *In vitro* cell safety and bioactivity

Cytotoxicity of AAM was investigated using HaCaT cells purchased from Istituto Zooprofilattico Sperimentale della Lombardia e dell’Emilia Romagna (Brescia, Italy). The culture conditions for HaCaT cells were the same as those used in our previous study (Yang et al. 2022). To determine the cell viability, 1.2 × 10^4^ cells/well were seeded in 96-well plates. After 24 h, a series of concentrations of raw AAM and ANF-2% in phosphate-buffered saline (PBS) was added and co-incubated with cells for another 24 h. Finally, the samples were removed and 150 µL/well of 0.5% MTT solution was added; 3 h later, the OD at 550 nm was measured using a microplate spectrophotometer (SpectraMax ABS Plus, Molecular Devices, San Jose, CA, USA), and cell viability was calculated using the following formula:

$$\mathrm{Cell viability}\;\left(\%\right)=\frac{{\mathrm{OD}}_{\text{sample}}}{{\mathrm{OD}}_{\text{control}}}\times 100\%$$

Cellular uptake of AAM was evaluated by incubating 4 × 10^5^ HaCaT cells in 12-well plates with AAM for 3, 6, 9, and 24 hours, followed by HPLC analysis of the cell lysates to determine the intracellular AAM content.

Intracellular reactive oxygen species (ROS) generation was detected using the 2',7'-dichlorodihydrofluorescein diacetate (H_2_DCFDA) assay. Briefly, cells with density of 1.2 × 10^4^ cells/well were pre-treated with samples for 3 hours, and incubated with 10 µM DCFH-DA for 30 min at 37 °C. After washing twice with PBS, the cells were exposed to PM suspension (50 μg/cm²) for 30 min, and the fluorescence intensity was measured at 485/535 nm using a fluorescence spectrophotometer (BioTek, Winooski, VT, USA). Protein analysis, including the expression of inflammatory and aging markers, was performed via Western blotting. The detailed procedures for these bioactivity assays have been previously described (Yang et al. 2022). Briefly, HaCaT cells were pretreated with samples and exposed to particulate matter (PM). Total cellular proteins were extracted, quantified, and analysed by SDS-PAGE and Western blotting to evaluate inflammation- and oxidative stress-related protein expression.

### Statistical analysis

All results, including HPLC quantification and encapsulation efficiency measurements, were obtained in triplicate (*n* = 3) and are expressed as mean ± standard deviation (SD). Significant differences among samples were determined by one-way analysis of variance (ANOVA) followed by Tukey’s post hoc test using SPSS version 20 (IBM, Armonk, NY, USA). A *p*-value of < 0.05 was considered statistically significant.

## Results

### Physical and chemical characterisation

#### Water solubility, yield, and entrapment efficiency

As showed in Table 2, the water solubility of the raw AAM was 4.31 ± 0.23 µg/mL. In contrast, all ANF formulations had an aqueous solubility greater than 600 µg/mL, indicating that the nanofiber formulations enhanced the water solubility of AAM by more than 150-fold. The yields of ANFs were approximately 76-89%, and the entrapment efficiency of all ANFs exceeded 99%. These results indicate that electrospinning enabled efficient incorporation of AAM into the nanofiber matrix with high encapsulation efficiency, providing a suitable platform for improving solubility of poorly water-soluble actives.

**Table 2.** The water solubility, yield and entrapment efficiency of raw AAM and ANFs.

	Water solubility (µg/mL)	Yield (%)	Entrapment efficiency (%)
Raw AAM	4.31 ± 0.23^b^	-	-
ANF-2%	651.59 ± 31.2^a^	89.11 ± 7.97^a^	> 99
ANF-4%	663.63 ± 11.97^a^	81.11 ± 9.45^a^	> 99
ANF-6%	667.35 ± 3.11^a^	76.13 ± 0.70^a^	> 99

Note: Results were expressed as mean ± standard deviation, all data were from triplicated experiments (*n* = 3). Data with different superscript alphabets in the same column indicate that they are statistically different (*p* < 0.05).

#### Surface morphology and fibre diameter

**Figure 1.**
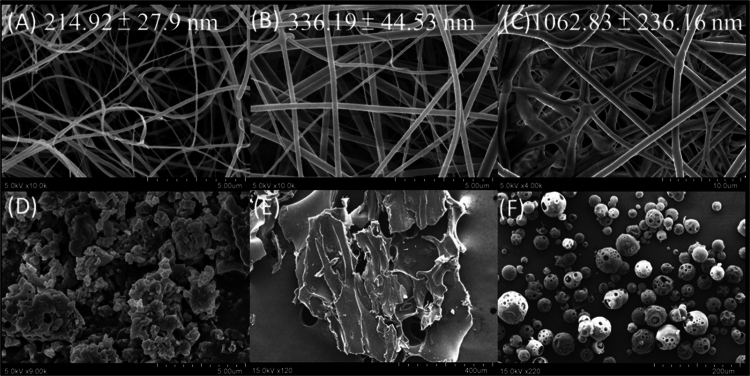
Surface morphology of (A) ANF-2%, (B) ANF-4%, (C) ANF-6%, (D) raw AAM, (E) PVP K120 and (F) HPBCD as observed by SEM.

Figure 1 panels A−C show the morphology of AAM nanofibers and Figure 1 panels D−F show the raw materials under SEM observation. The clump-like AAM, sheet-like PVP, and spherical HPBCD were converted into uniform nanofibers after electrospinning. Under optimised electrospinning conditions, smooth, bead-free, and homogeneous nanofibers were obtained. The diameter of ANF-2%, ANF-4%, and ANF-6% were calculated to be 214.92 ± 27.9, 336.19 ± 44.53, and 1062.83 ± 236.16 nm, respectively. This observation indicates that increasing the ratio of PVP in the polymer solution resulted in thicker fibres. The viscosity of the polymer solution thus affects the diameter of the fibres; that is, the higher the viscosity, the thicker the fibre produced.

#### Particle size analysis

**Figure 2.**
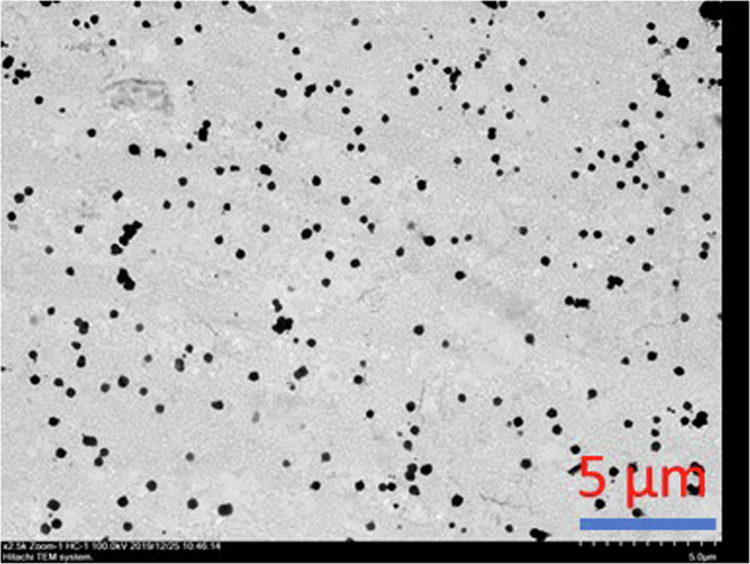
Transmission electron microscopy images of ANF-2% dissolved in water.

TEM analysis of ANF-2% in aqueous solution revealed spherical nanoparticles (~500 nm) (Figure 2). Dynamic light scattering (DLS) measurements confirmed a particle size of 507.67 ± 14.93 nm with a PDI of 0.328. Increasing PVP content significantly reduced particle size to 288.67 ± 14.81 nm (ANF-4%) and 213.40 ± 5.88 nm (ANF-6%), with corresponding decreases in PDI values (*p* < 0.05) (Table 3). These findings suggest that higher PVP ratios improved dispersion and colloidal stability, which are desirable for topical formulations.

**Table 3.** The particle size of ANFs.

	Particle size (nm)	Polydispersity index (PDI)
ANF-2%	507.67 ± 14.93^a^	0.328 ± 0.011^a^
ANF-4%	288.67 ± 14.81^b^	0.210 ± 0.006^b^
ANF-6%	213.40 ± 5.88^c^	0.160 ± 0.004^c^

Note: Values are the mean ± SD of three independent experiments (*n* = 3). Data with different superscript alphabets in the same column indicate that they are statistically different (*p* < 0.05).

#### Crystallinity and molecular interactions

**Figure 3.**
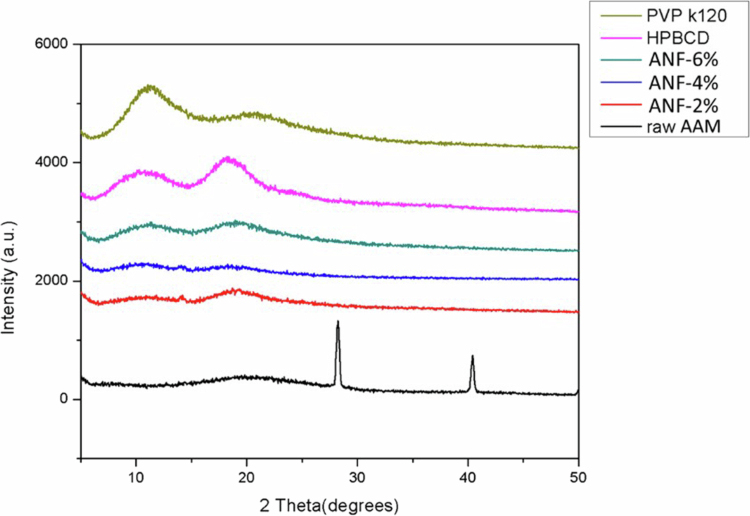
The X-ray diffraction patterns of PVP K120, HPBCD, different ratio ANFs, and raw AAM.

To investigate the crystalline state and molecular interactions, X-ray diffraction (XRD) and Fourier-transform infrared spectroscopy (FT-IR) spectroscopy analyses were performed. As shown in Figure 3, the diffraction pattern of numerous peaks indicated a highly crystalline active compound. The amorphous state of the plant extract was confirmed by the disappearance of the characteristic peaks. XRD patterns of raw AAM displayed characteristic crystalline peaks at 28.2° and 40.4°. These peaks disappeared in ANFs, showing a broad diffuse halo across the scanned range, which confirmed the successful transformation of AAM into an amorphous state within the polymeric matrix (Figure 3). This shift is crucial for enhancing the water solubility and bioavailability of AAM.

**Figure 4.**
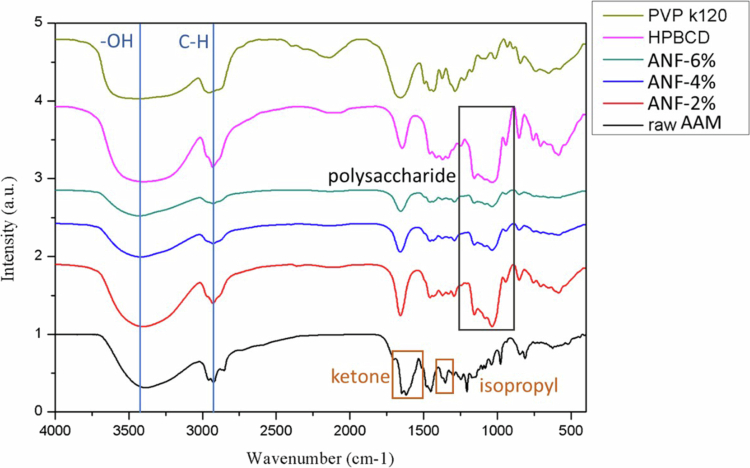
FTIR spectrum of AAM, AAM nanofibers, HPBCD and PVP K120.

The FT-IR spectra further demonstrated molecular interactions of the raw AAM, ANFs, and excipients in Figure 4. Raw AAM exhibited distinct ketone peak at 1618 cm^–1^ and stretching vibration peak of isopropyl appeared at 1353 cm^–1^. In the HPBCD and ANF spectrum, a broad ─OH stretching vibration absorption was observed near 3390 cm^–1^ and the absorption bands of polysaccharide were observed near 1034 cm^–1^. In the case of the ANFs, the characteristic peak of AAM could not be detected, and the polysaccharide absorption band was clear, which might be attributed to the shielding of the characteristic absorption peaks of AAM, as they were included in the CD cavity. In summary, these results indicated that electrospinning successfully transformed AAM from crystalline to amorphous state and stabilised it within the polymeric matrix.

### Antioxidant ability

**Figure 5.**
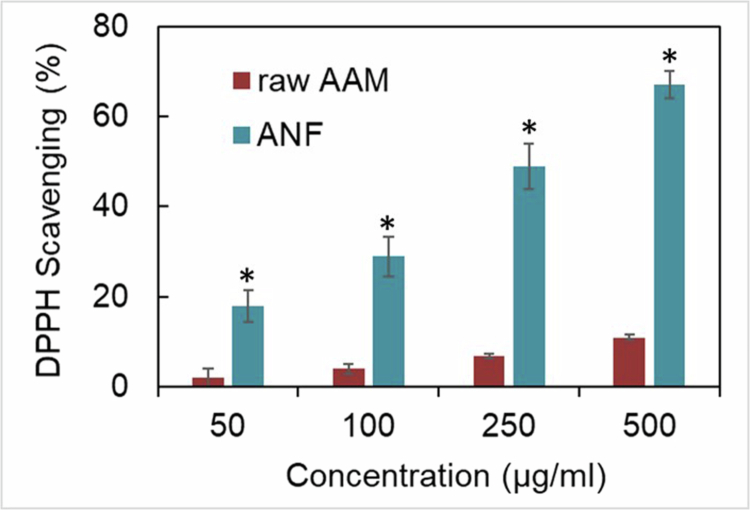
DPPH free radical scavenging potential of raw AAM suspension and ANF-2% dissolved in deionized water. Values represent mean ± SD (*n* = 3). * represents *p* < 0.05 when compared to raw AAM.

Proper pharmaceutical formulation and design should not only encapsulate the active ingredient into the excipients but also possess the ability to release the active ingredient. The present study used a common free radical scavenging assay, the DPPH free radical scavenging assay, to verify whether AAM could be released from the nanofibers and exert antioxidant activity. Figure 5 clearly shows that raw AAM suspended in deionized water showed weak scavenging activity. However, after encapsulation into nanofibers, ANF-2% provided significantly greater scavenging ability, likely because of the better aqueous solubility of ANF compared with raw AAM (*p* < 0.05).

### *Ex* vivo skin penetration ability

The skin penetration abilities of the ANFs and raw AAM suspensions were investigated by *ex vivo* tests over 0.5, 1, 2, and 4 h on porcine skin were showed in Table 4. The epidermal permeability of raw AAM in the nanofibers was significantly improved when encapsulated in HPBCD/PVP (*p* < 0.05). Our results revealed that the raw AAM suspension had little drug penetration (almost zero) for up to 4 h compared with ANF-2% with drug penetration at 11.5 ± 0.69−42.66 ± 6.78 µg/cm^2^. The level of ANF-2% was 145-fold higher than that of raw AAM, and the absorption ANF-2% occurred in a time-dependent manner in the epidermis. The skin permeation data revealed a steady-state flux (Jss) of 8.47 µg/(cm^2^·h) for the ANF-2%, which was significantly higher than the 0.0835 µg/(cm^2^ ⋅h) observed for raw AAM. This represents an enhancement ratio of approximately 101-fold. The corresponding permeability coefficients (Kp) were calculated to be 8.47 × 10^⁻3^ cm/h for ANF-2% and 8.35 × 10^−5^ cm/h for raw AAM, based on a donor concentration (Cd) of 1000 µg/mL.

**Table 4.** The *Ex vivo* percutaneous permeation of raw AAM and ANF through porcine skin.

	Time (h)	*Stratum corneum* (µg/cm^2^)	Epidermis (µg/cm^2^)	Dermis (µg/cm^2^)
Raw AAM	0.5	0.06 ± 0.01	0.11 ± 0.15	N.D.
	1	0.08 ± 0.02	0.17 ± 0.19	N.D.
	2	0.04 ± 0.01	0.22 ± 0.13	N.D.
	4	0.20 ± 0.10	0.28 ± 0.14	N.D.
ANF-2%	0.5	5.29 ± 0.11*	10.83 ± 1.23*	0.67 ± 0.31
	1	0.98 ± 0.32*	30.22 ± 4.51*	0.43 ± 0.12
	2	12.5 ± 4.85*	28.35 ± 2.47*	1.56 ± 0.61
	4	7.89 ± 4.56*	40.74 ± 3.12*	1.92 ± 0.52

Note: Values represent mean ± SD of five independent experiments (*n* = 5). N.D.: Not detected; * represents *p* < 0.05 when compared to raw AAM.

Both formulations showed no measurable lag phase, consistent with immediate drug release. The results showed that the formulation burst release phase occurred within 1 h owing to drug deposition and release from the surface of the nanofibers. However, after 1 h, the release of the drug from the nanofibers was retarded, which represented a sustained release pattern. The sustained release with an initial burst phase of ANF-2% may provide sufficient bioactivity of the drug in the skin.

### *In* vitro cell assay

#### Cell safety

**Figure 6.**
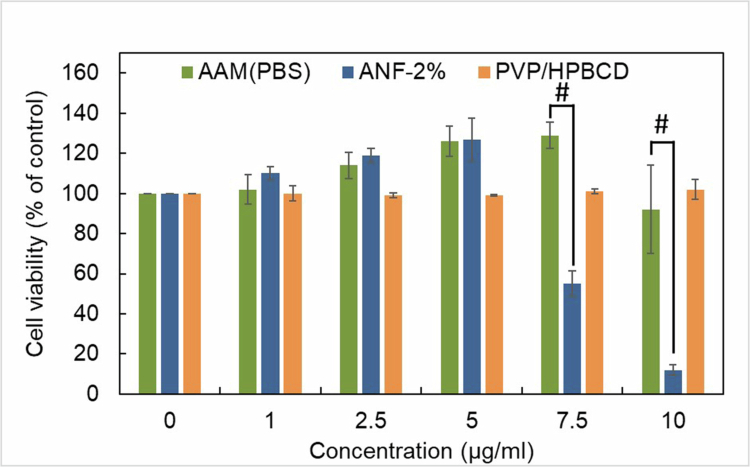
Effects of AAM, ANF-2% and excipients on HaCaT keratinocytes viability. All values are the mean ± SD of three independent experiments (*n* = 3). ^#^ were considered significantly different (*p* < 0.05) between AAM (PBS) and ANF-2% group.

It is important to evaluate the biocompatibility of pharmaceutical ingredients with the human body to avoid cytotoxicity. Therefore, toxicological evaluation was performed according to ISO10993-5 guidelines. Figure 6 demonstrates the cell viability of HaCaT keratinocytes treated with raw AAM, ANF-2%, and excipients. No cell death was observed with raw AAM or excipient treatment at concentrations less than 10 µg/mL. However, reduction in cell viability was found when the cells were treated with ANF-2% at concentrations above 7.5 µg/mL (IC₅₀ ≈ 7.5 µg/mL). A dose-dependent reduction in viability likely attributable to the enhanced aqueous solubility of AAM within ANF-2%, which facilitated greater cellular uptake and intracellular accumulation. Consequently, a sufficient intracellular concentration of AAM was achieved to elicit cytotoxic responses. In other words, AAM was more easily absorbed by the cells after its preparation as nanofibers.

#### Cellular uptake

To further confirm the cellular absorption AAM and ANF-2% in HaCaT keratinocytes, we collected the cell lysates at four different timepoints to analyse the cellular AAM content (Table 5). Raw AAM (suspended in PBS) was not detected at any time point, indicating that raw AAM rarely entered the cells. In contrast, we found the presence of ANF-2% in cells, with the highest accumulation observed at 9 h (but not significantly different from other timepoints, *p* > 0.05). These results indicated that ANF-2% significantly reduced the particle size of AAM (*p* < 0.05), thereby increasing its interaction with the cell membrane and enhancing cellular uptake. This finding directly correlates the enhanced cellular penetration with the observed reduction in cell viability at higher concentrations.

**Table 5.** The intercellular uptake of ANF-2% and raw AAM.

	3 h	6 h	9 h	24 h
Raw AAM	N.D.	N.D.	N.D.	N.D.
ANF-2%	1.17 ± 0.33	1.34 ± 0.45	2.0 ± 0.40	1.57 ± 0.33

Note: Values are the mean ± SD of three independent experiments (*n* = 3). N.D.: Not detected.

#### Reduction of intracellular ROS generation

**Figure 7.**
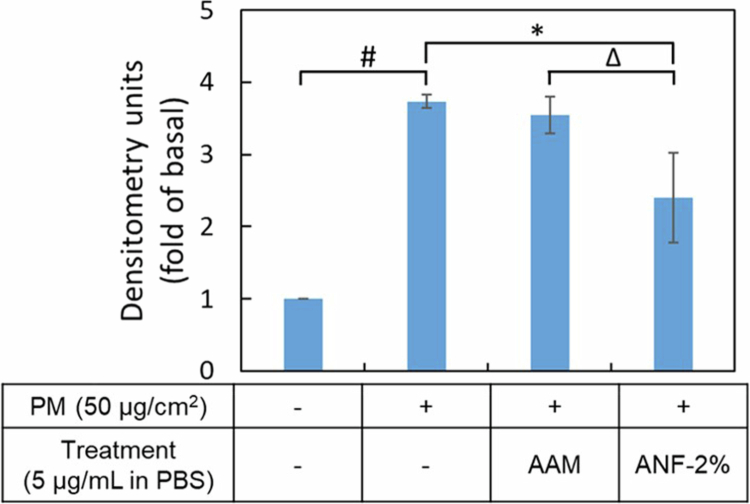
Effects of AAM and ANF-2% on PM-induced ROS generation in HaCaT cells. The intracellular ROS levels were observed by a fluorescent spectrometer. Values are the mean ± SD of three independent experiments (*n* = 3). * represents *p* < 0.05 when compared to raw AAM; ^∆^ represents *p* < 0.05 when compared to AAM PBS group.

As shown in Figure 7, exposure to particulate matter (PM) significantly induced oxidative stress in HaCaT keratinocytes by nearly 4-fold increase in intracellular reactive oxygen species (ROS) generation compared to the untreated control group (*p* < 0.05). Treatment with raw AAM (5 µg/mL, suspended in PBS) showing a mere 7% inhibition of ROS production. In contrast, at the same concentration of ANF-2% substantially inhibited ROS generation by 48.8% (*p* < 0.05). These results demonstrate that the ANF-2% formulation provides superior antioxidant activity. This enhanced efficacy is directly linked to its dramatically improved water solubility, which facilitates greater bioavailability and subsequent cellular uptake of the extract, thereby allowing for a more potent intracellular antioxidant response.

#### Inhibition of PM-induced COX-2 inflammatory protein and MMP-1 aging protein expression

**Figure 8.**
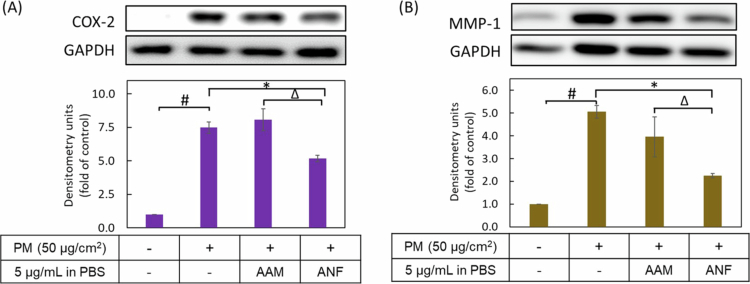
Effects of AAM and ANF-2% on PM-induced inflammation and skin aging. Protein expression of (A) COX-2 and (B) MMP-1 were analysed by Western blotting and densitometry. Values are the mean ± SD of three independent experiments (*n* = 3). ^#^ represents *p* < 0.05 when compared to untreated group (control group); * represents *p* < 0.05 when compared to PM treatment alone group (PM group); ^∆^ represents *p* < 0.05 when compared to AAM PBS group.

To compare the anti-inflammatory and anti-aging effects of the raw AAM and ANF-2% formulation, the protein expression of key biomarkers was evaluated using Western blotting (Figure 8). HaCaT cells exposed to 50 µg/cm^2^ PM significantly upregulated the expression of cyclooxygenase-2 (COX-2) and matrix metalloproteinase-1 (MMP-1) by 7.5-fold and 4.6-fold, respectively, relative to the untreated control. Pretreatment with the ANF-2% formulation at a concentration of 5 µg/mL significantly suppressed COX-2 expression by 35.5% and MMP-1 expression by 69%. However, pretreatment with the raw AAM extract, applied as a suspension in PBS, had no significant inhibitory effect on the expression of either protein. ANF-2% formulation exerts potent anti-inflammatory effects by reducing COX-2 protein expression and provides significant anti-aging benefits by inhibiting MMP-1 expression in PM-stimulated HaCaT keratinocytes. This superior bioactivity is directly correlated with the enhanced cellular bioavailability of AAM facilitated by the nanofiber delivery system.

#### Downregulation of the MAPK signalling pathway

**Figure 9.**
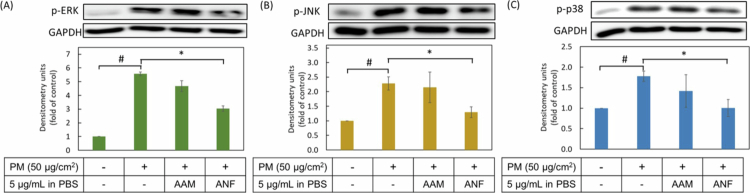
Effects of AAM and ANF-2% on PM-induced MAPK phosphorylation. Protein expression of (A) *p*-ERK, (B) *p*-JNK and (C) *p*-p38 were analysed by Western blotting and densitometry. Values are the mean ± SD of three independent experiments (*n* = 3). ^#^ represents *p* < 0.05 when compared to untreated group (control group); * represents *p* < 0.05 when compared to PM treatment alone group (PM group).

The phosphorylation of mitogen-activated protein kinases (MAPKs), including ERK, JNK, and p38, is a well-established cellular response to environmental stressors such as particulate matter (PM). This phosphorylation cascade is known to mediate inflammatory and skin aging responses. As shown in Figure 9, PM exposure significantly increased the phosphorylation of ERK, JNK, and p38 in HaCaT keratinocytes, confirming the successful induction of the signalling pathway. Pretreatment with the ANF-2% formulation at a concentration of 5 µg/mL potently and dose-dependently suppressed the phosphorylation of ERK, JNK, and p38 by 55.3%, 77%, and 99.6%, respectively. In stark contrast, no significant inhibitory effect was observed for the raw AAM extract (suspended in PBS), likely due to its deficient cellular uptake.

## Discussion

Polymeric nanofibers have been widely utilised to enhance the efficiency of low-bioavailability components, such as ciprofloxacin (Uhljar et al. 2021), paclitaxel (Chi et al. 2020), and ibuprofen (Celebioglu and Uyar 2019) by improving their aqueous solubility, biological barrier permeability, stability, dissolution rate, and targeted delivery. Electrospun nanofibers provide high loading efficiency, yield, narrow particle size distribution, simple production processes, and use small amounts of stabilisers during the preparation process. These nanofibers can be used to obtaining dry final products without time- and energy-consuming steps such as lyophilization (Adibkia et al. 2019). In this study, to overcome the poor skin penetration ability of AAM, we successfully prepared ANFs using electrospinning technology with a surfactant-free, continuous, and simple method.

The formation of the nanofibers depends on the concentration of the polymer in the feed solution. A low polymer content in the solution leads to insufficient viscosity, which may break the liquid jet into separated droplets and form spherical electrospray nanoparticles. In contrast, providing sufficient viscosity during the electrospinning process helps continuous jets to form instead of separated droplets (Coelho et al. 2022; Silva et al. 2022). It was found that a polymer solution containing 2%−6% of PVP K-120 provided excellent spinnability and exhibited favourable encapsulation efficiency, yield, and water solubility.

Obtaining uniform and thin fibres is the goal of the electrospinning process. SEM images as shown in Figure 1 were used to determine the morphology of the ANFs without the beads. Bead formation is considered insufficient for spinnability or unsuitable for manufacturing parameters, which may dictate the dispersion of the drug in the formulation. ANF may have improved the skin penetration capacity of AAM in the following ways: (i) reduction of particle size: Several studies have illustrated that a drug delivery system that can decrease particle size to increase surface area can achieve better skin penetration, such as resveratrol (Lin et al. 2020), tetrahydrocurcumin (Kakkar et al. 2018), and myricetin (Lin et al. 2023). In the current study, hydrophobic AAM entered the cavity of HPBCD to form an inclusion complex, followed by the addition of PVP by electrospinning. When ANF was dissolved in aqueous solution, PVP helped disperse the particles and form a homogenous colloidal system; therefore, the ANF particles were uniform under DLS analysis; (ii) transformation of crystallinity: The crystalline form of the drug molecule affects its dissolution and absorption properties. Insoluble drug molecules with high crystallinity could transform into an amorphous state through a solid dispersion manufacturing process. Lattice energy is the main obstacle to dissolving crystalline drug molecules. The amorphous state of a drug molecule thus possesses higher free energy to overcome the energy barrier, leading to better water solubility, dissolution rate, and absorption (Baghel et al. 2016). Patel et al. (2019) encapsulated raloxifene in a lipid matrix to form raloxifene-loaded solid lipid nanoparticles (RL-SLN). RL-SLN remarkably decreased the crystallinity of raloxifene, which provided better skin penetration ability of raloxifene (Patel et al. 2019); (iii) intermolecular interaction: The drug molecule may connect with excipients (polymers) through various weak forces, such as H-bonding, van der Waals forces, or electrostatic interactions. These bonds limit the mobility of drug molecules in solid dispersion without changing the major structure of the drug molecule. In addition, weak forces play an important role in maintaining the stability of formulations and preventing drug recrystallisation (Meng et al. 2015). Browne et al. (2020) established that the presence of PVP in the formulation may increase the stability of the amorphous state and aqueous solubility of ketoprofen (Browne et al. 2020).

Moreover, the occlusive effect decreases transepidermal water loss, which may affect skin hydration status and barrier permeability. Increasing the skin hydration ability is a noninvasive method for enhancing drug molecule penetration without disrupting the barrier function. The hydration effect does not significantly change the layer structure of the skin tissue but causes swelling of the corneocytes (*p* > 0.05). The swollen corneocytes disrupt the lipid order to form a spectrum of disorganised lacunae in the intercellular regions. Thus, improved skin hydration is required for drug penetration (Chauhan 2017; Warner et al. 2003; Zhai and Maibach 2001). In a previous study, Saini et al. (2022) improved skin penetration ability of tetrahydrocurcumin through formation of an adhesive layer on the skin surface to provide an occlusive effect using tetrahydrocurcumin lipid nanoparticle-based gel (THC-SLNs) (Saini et al. 2022). In the current study, ANF improved the water solubility of raw AAM by 150-fold and penetration by 145-fold compared with raw AAM as shown in Table 2. The increased skin penetration ability of ANF may be attributed to the excellent film-forming ability of PVP. When the ANF particles were deposited on the skin surface, a film-like layer was formed, which caused an occlusive effect on the skin and enhanced the water-retention capacity (Guo et al. 2011; Kumar et al. 2014; Pünnel and Lunter 2021). Therefore, skin hydration promoted the penetration of AAM deeper into the epidermis and dermis.

**Figure 10.**
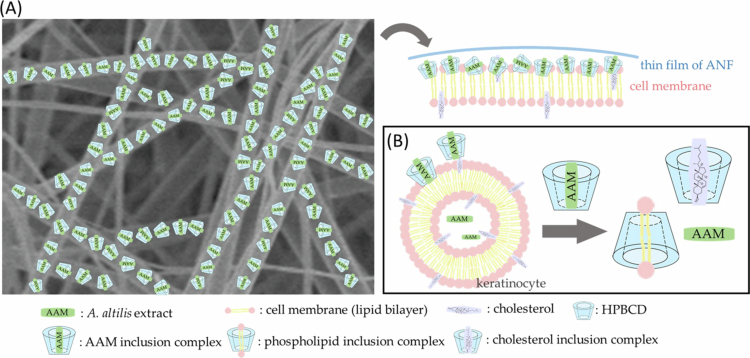
A proposed cellular uptake mechanism of AAM in keratinocytes.

Only if the active ingredients successfully penetrate the stratum corneum can they reach the epidermis and dermis. ANF could enter keratinocytes in the viable epidermis and express its bioactivity adequately. CDs can selectively include lipophilic components into the hydrophobic cavity such as phospholipid bilayer and cholesterol (López et al. 2011). CDs do not bind to specific receptors on the cell membrane but react in a non-classical manner. Hammoud et al. (2014) reviewed the interactions between CDs and biomimetics or cell membranes. This interaction is based on extraction and exchange mechanisms (Hammoud et al. 2019). The components of the cell membrane provide better affinity to CDs than the AAM. Thus, when the AAM-HPBCD complex attached to the cell membrane, extraction and exchange occurred. In other words, CDs prefer to incorporate cholesterol; similarly, AAM was released and delivered into the cells (Figure 10).

Penetration of AAM to the deep skin layer and been intake affect the response of cells to the external environment (Chen Chen et al. 2018). The outcome is attributed to that electrospun nanofibers completely retained the pharmacological activity of AAM. The interaction with HPBCD and PVP did not block the release of AAM. In this study, ANF reducing ROS generation, and inhibiting the phosphorylation of MAPK signalling pathway (ERK, JNK and p38) to possess anti-inflammation (COX-2) and anti-aging (MMP-1) bioactivities. Finally, ANF expressed more excellent anti-pollutant activity than raw AAM.

## Conclusions

Present study demonstrated that electrospun PVP/HPBCD nanofibers provide an effective topical delivery platform for *Artocarpus altilis* methanolic extract (AAM). The nanofiber formulation improved the aqueous solubility and amorphous dispersion of AAM, resulting in enhanced skin permeability, greater cellular uptake, and superior antioxidant and anti-inflammatory activity compared with the raw extract. These findings confirm the pharmaceutical importance of nanofiber-based delivery systems in overcoming solubility and permeability limitations of hydrophobic compounds. Beyond AAM, this approach underscores the potential of electrospun nanofibers as a versatile and biocompatible strategy for improving the solubility, permeability, and controlled release of poorly water-soluble bioactives, offering broad applicability in topical formulation engineering for both therapeutic and cosmetic drug delivery.

## Supplementary Material

Supplementary data.docx Supplementary data.docx

raw data western blot.pptx raw data western blot.pptx

## Data Availability

The datasets in current study are available on request.
