# Supplementary material for: Electrospun PVP/HPBCD nanofiber topical drug delivery platform for enhanced skin permeability and anti-pollution bioactivity of Artocarpus altilis extract
Source: Drug Deliv. 2026 Jan 6;33(1):2610654. doi: 10.1080/10717544.2025.2610654 (PMC12781955; doi:10.1080/10717544.2025.2610654)
Supplement: Supplementary data.docx [file IDRD_A_2610654_SM7259.docx]

**Extraction Yield and Quantification of Artocarpin in AAM**

The extraction yield of AAM was about 2.8%. As showed in Figure S1(A), the index component, artocarpin, gave a characteristic peak at 7.2 min. In Figure S1(B), the AAM chromatography also displayed an obviously peak at same retention time (7.2 min), which could be identified as artocarpin. The artocarpin content of AAM used in present study was 181 μg artocarpin in per mg of AAM. It was very important to quantify the amount of index component in batch of AAM since the amount of artocarpin may affect the characteristics and the cytotoxicity of AAM.


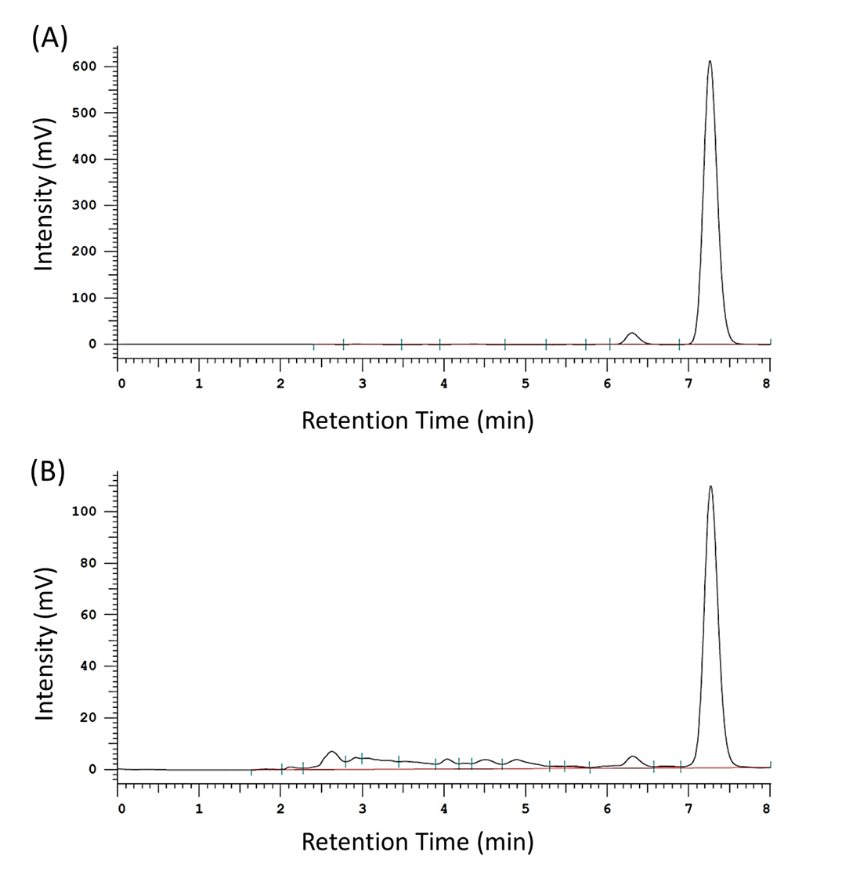


Figure S1. HPLC profile of (A) index component, artocarpin (100 μg/mL) and (B) *Artocarpus atilis* methanolic extract (100 μg/mL)
