# Supplementary material for: Electrospun PVP/HPBCD nanofiber topical drug delivery platform for enhanced skin permeability and anti-pollution bioactivity of Artocarpus altilis extract
Source: Drug Deliv. 2026 Jan 6;33(1):2610654. doi: 10.1080/10717544.2025.2610654 (PMC12781955; doi:10.1080/10717544.2025.2610654)
Supplement: raw data western blot.pptx [file IDRD_A_2610654_SM7258.pptx]

## Slide 1
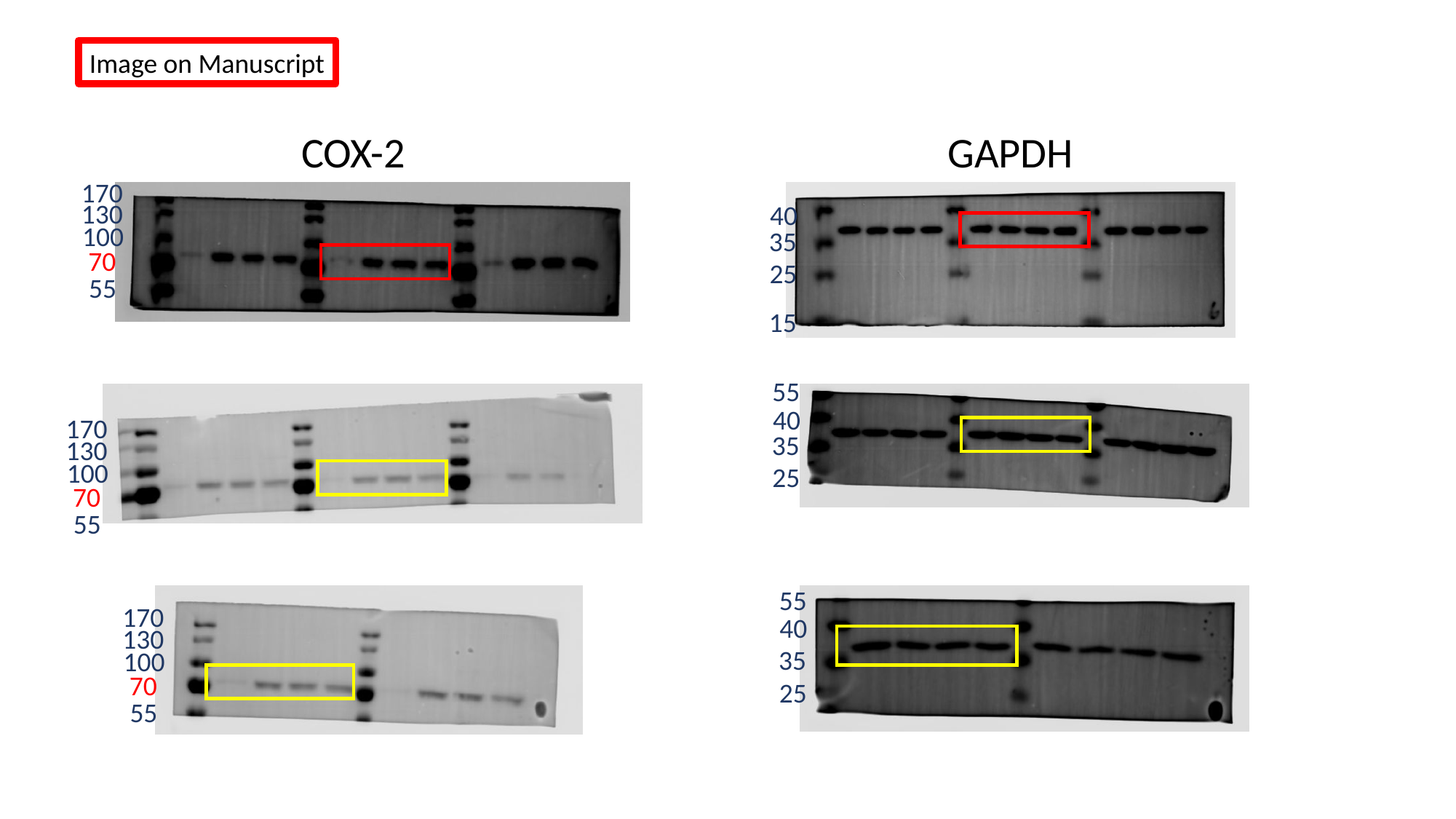

Image on Manuscript
COX-2
GAPDH
170
130
40
100
35
70
25
55
15
55
40
170
35
130
100
25
70
55
55
170
40
130
35
100
70
25
55

## Slide 2
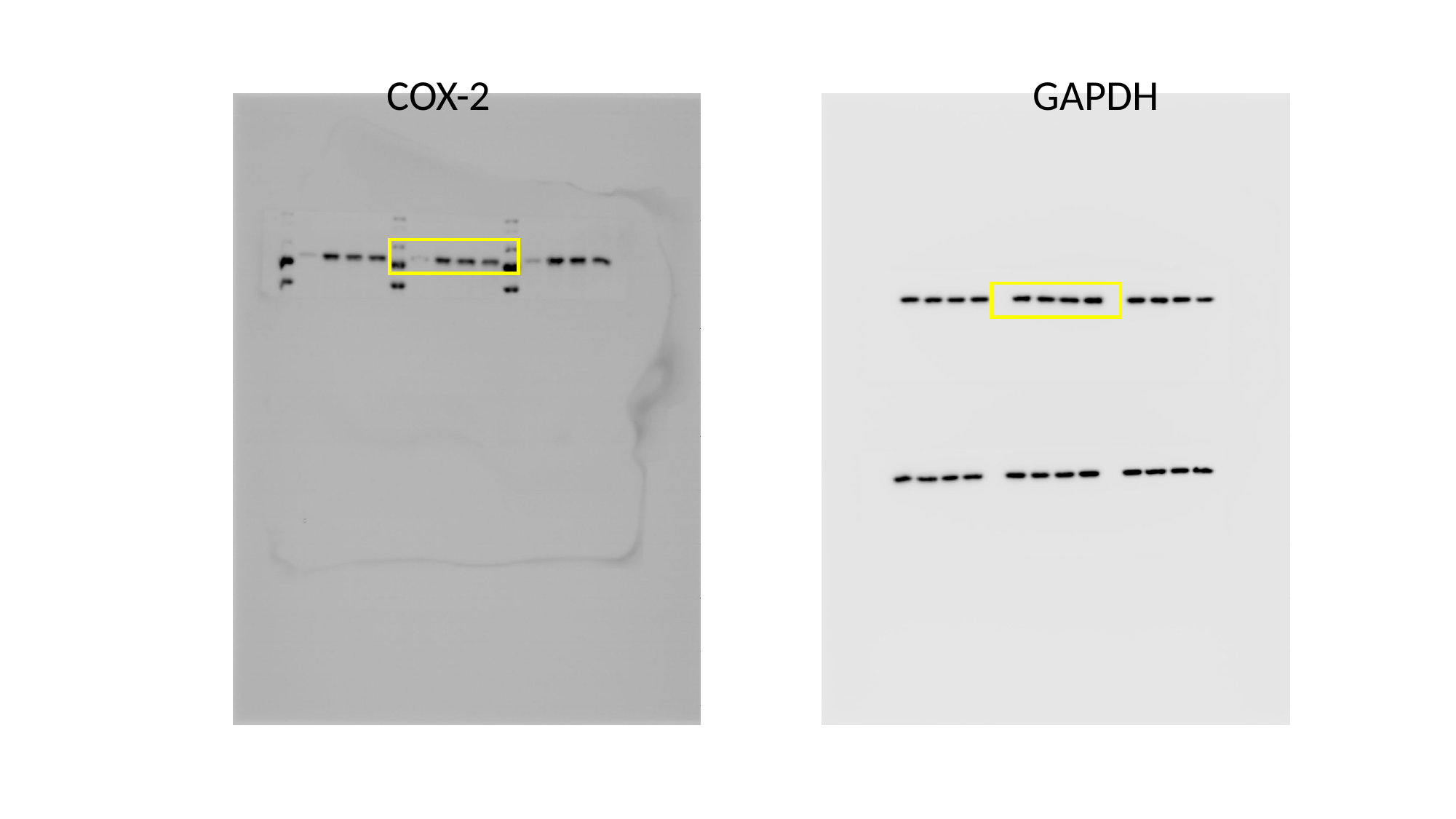

COX-2
GAPDH

## Slide 3
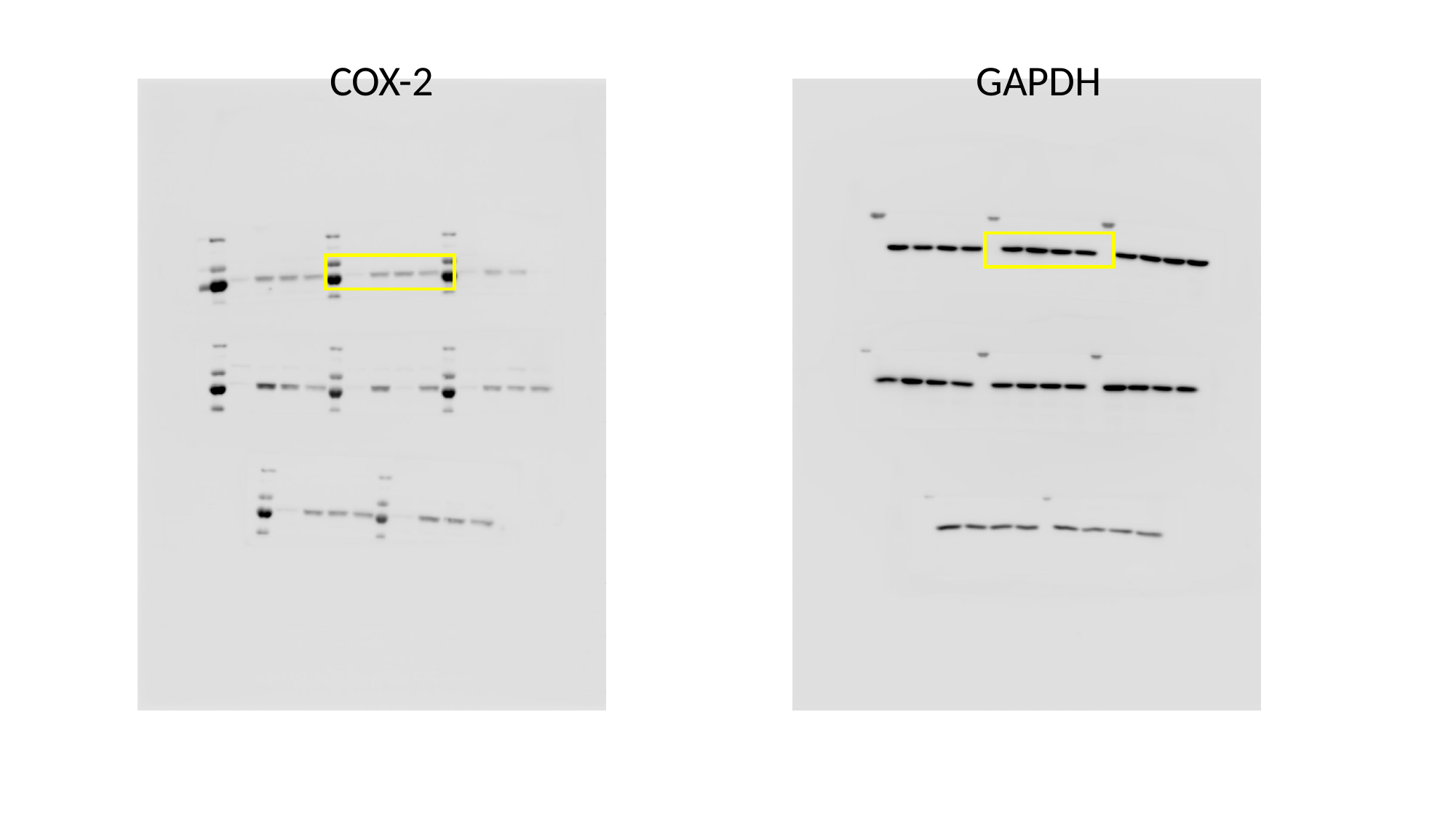

COX-2
GAPDH

## Slide 4
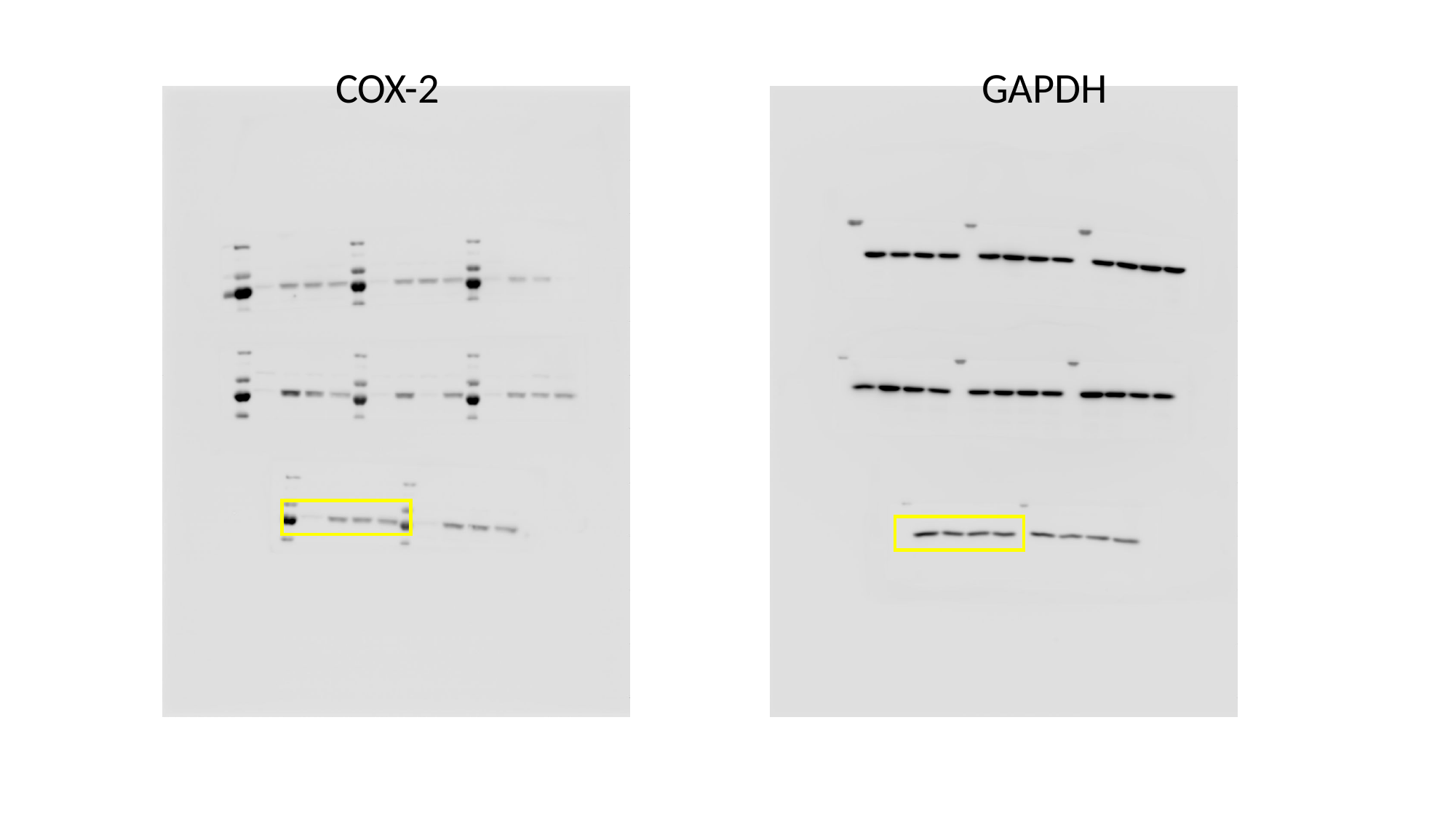

COX-2
GAPDH

## Slide 5
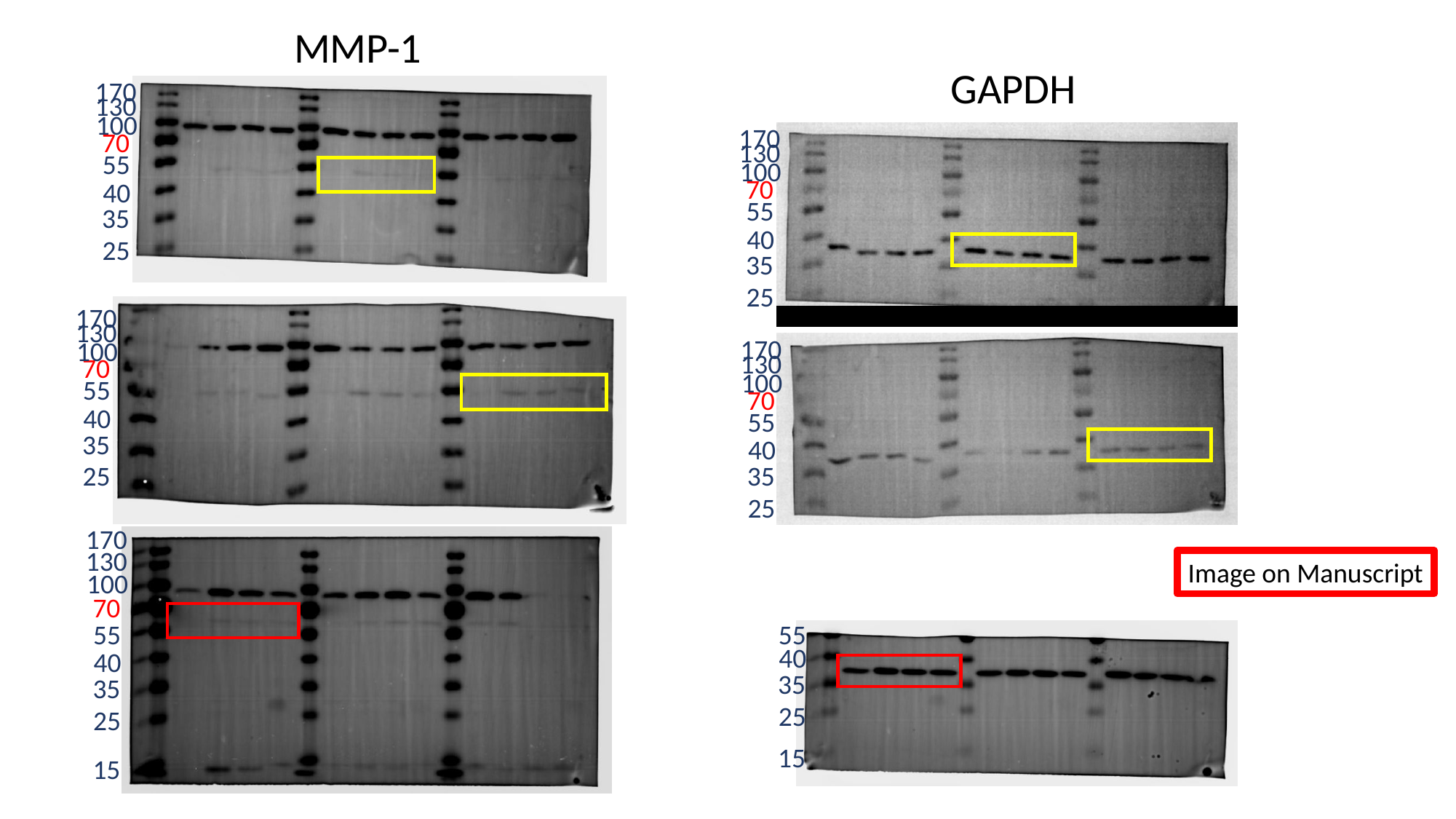

MMP-1
GAPDH
170
130
100
170
70
130
55
100
70
40
55
35
40
25
35
25
170
130
170
100
130
70
100
55
70
40
55
35
40
35
25
25
170
130
Image on Manuscript
100
70
55
55
40
40
35
35
25
25
15
15

## Slide 6
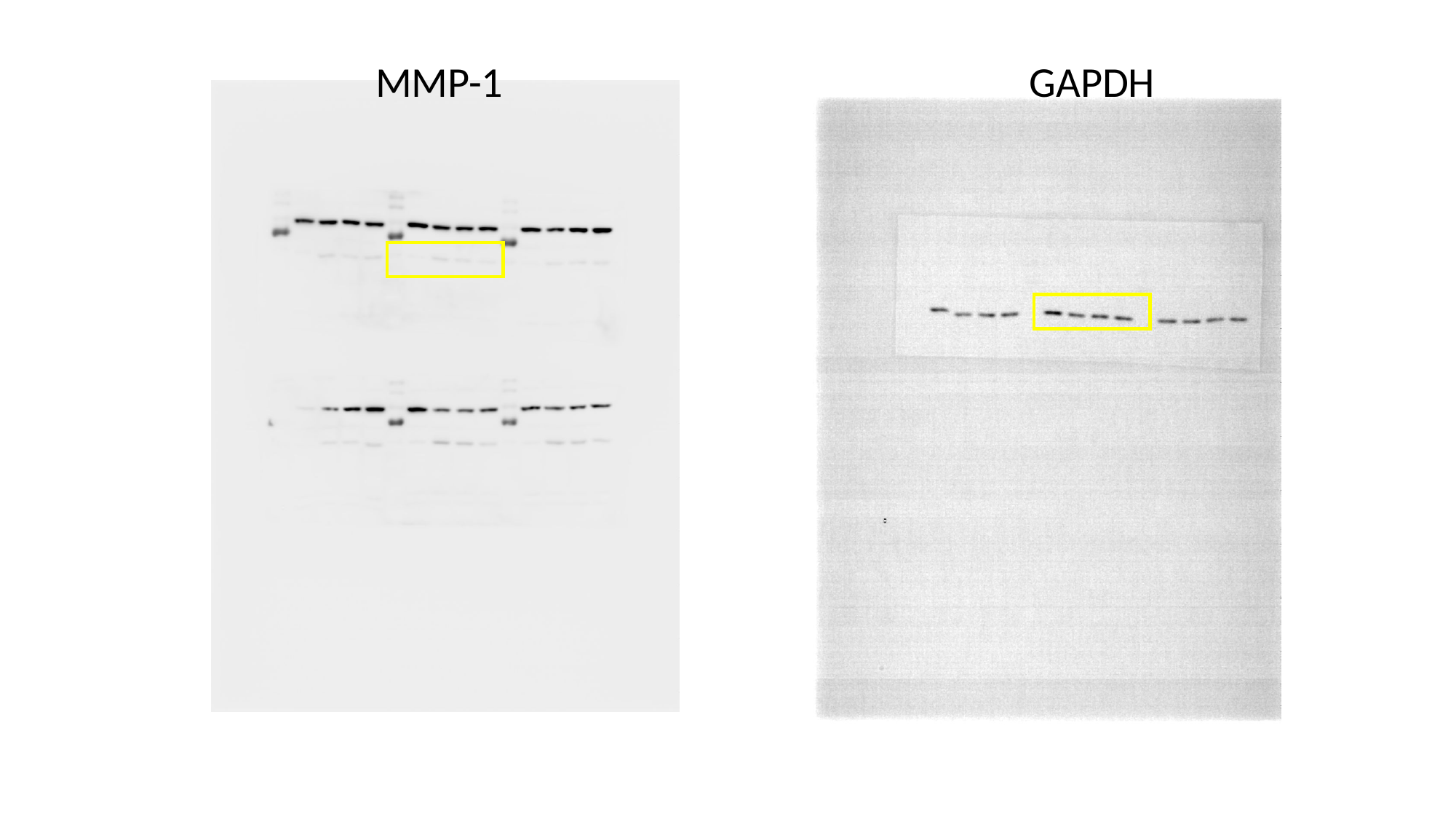

MMP-1
GAPDH

## Slide 7
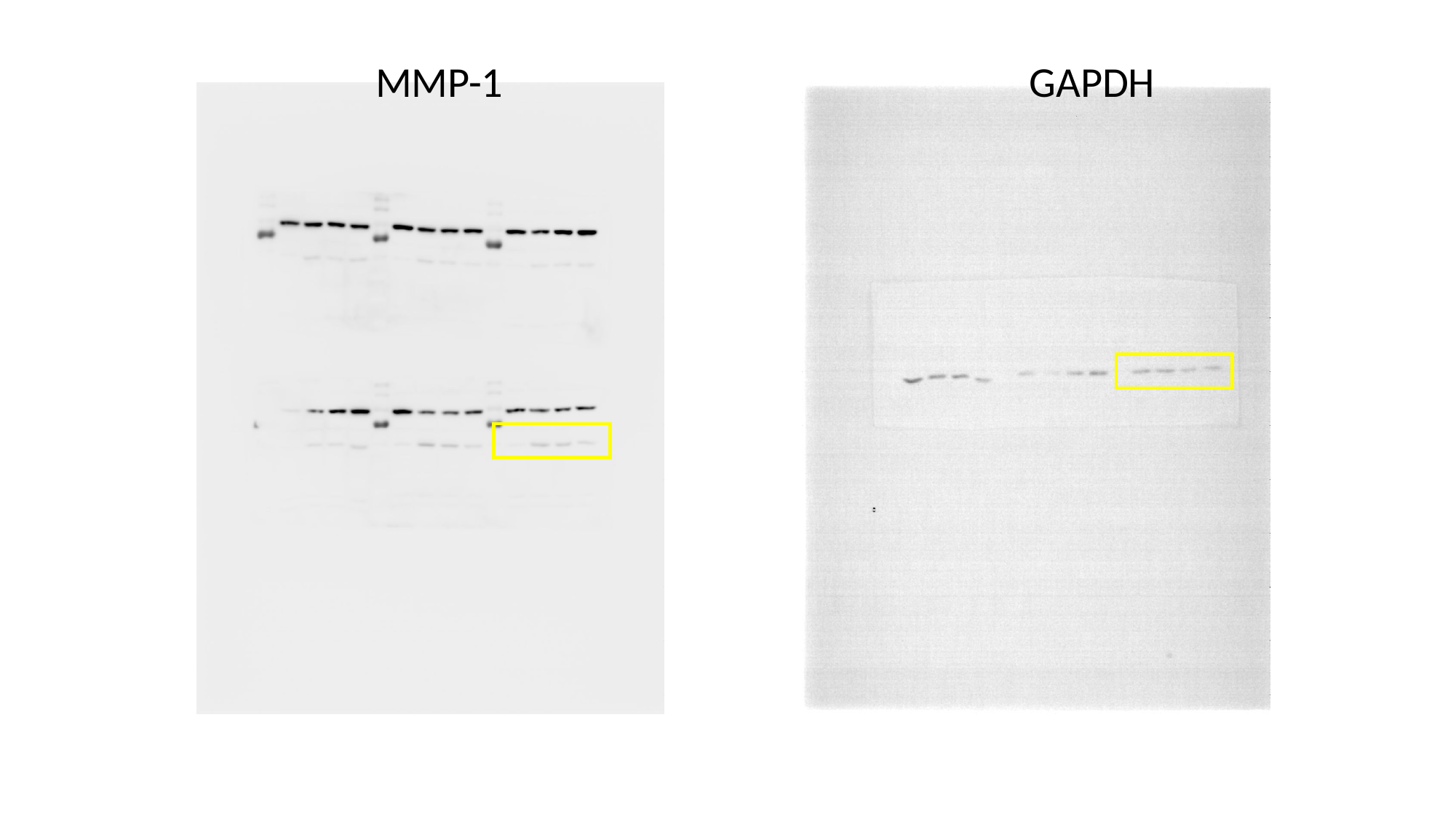

MMP-1
GAPDH

## Slide 8
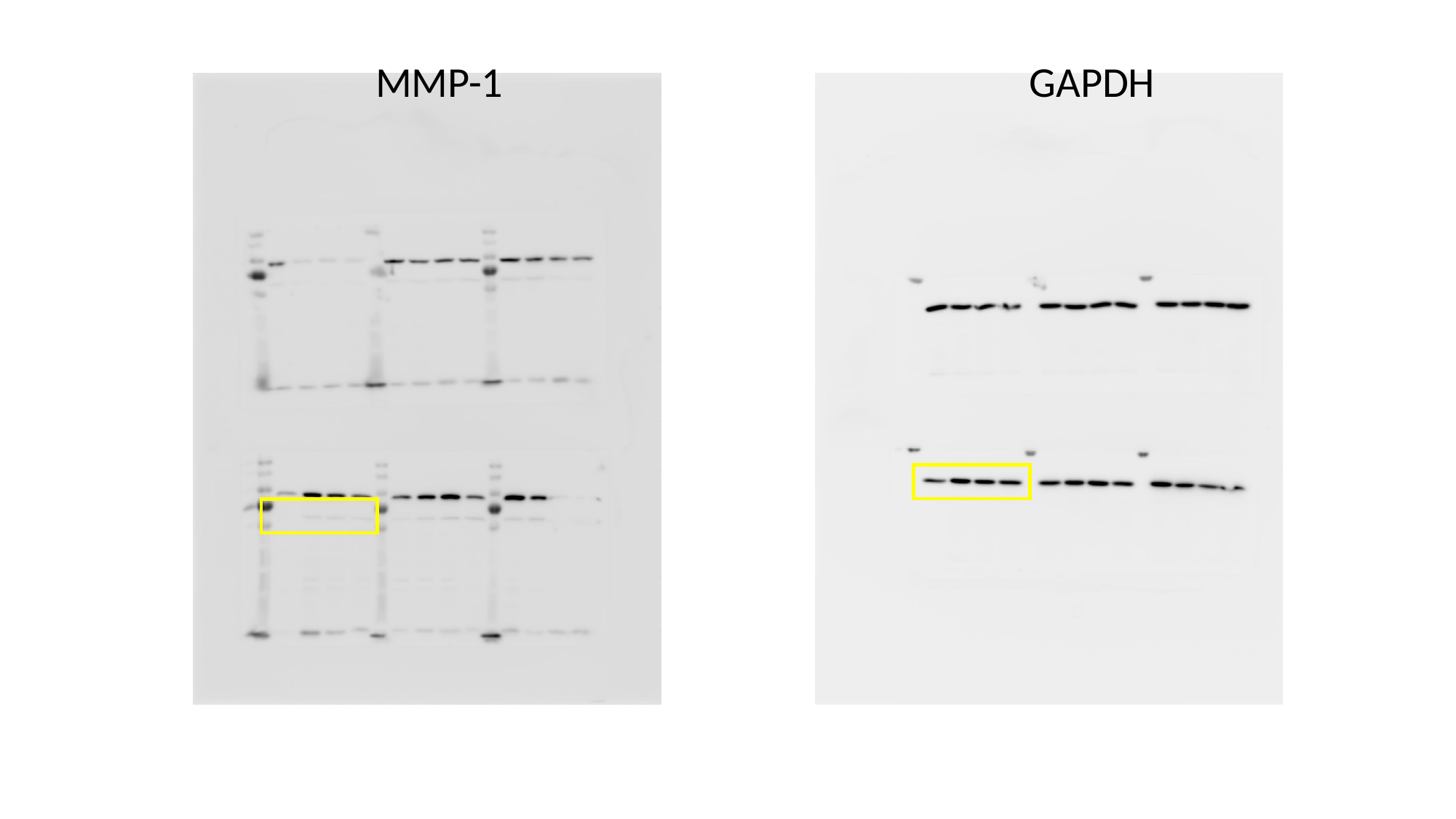

MMP-1
GAPDH

## Slide 9
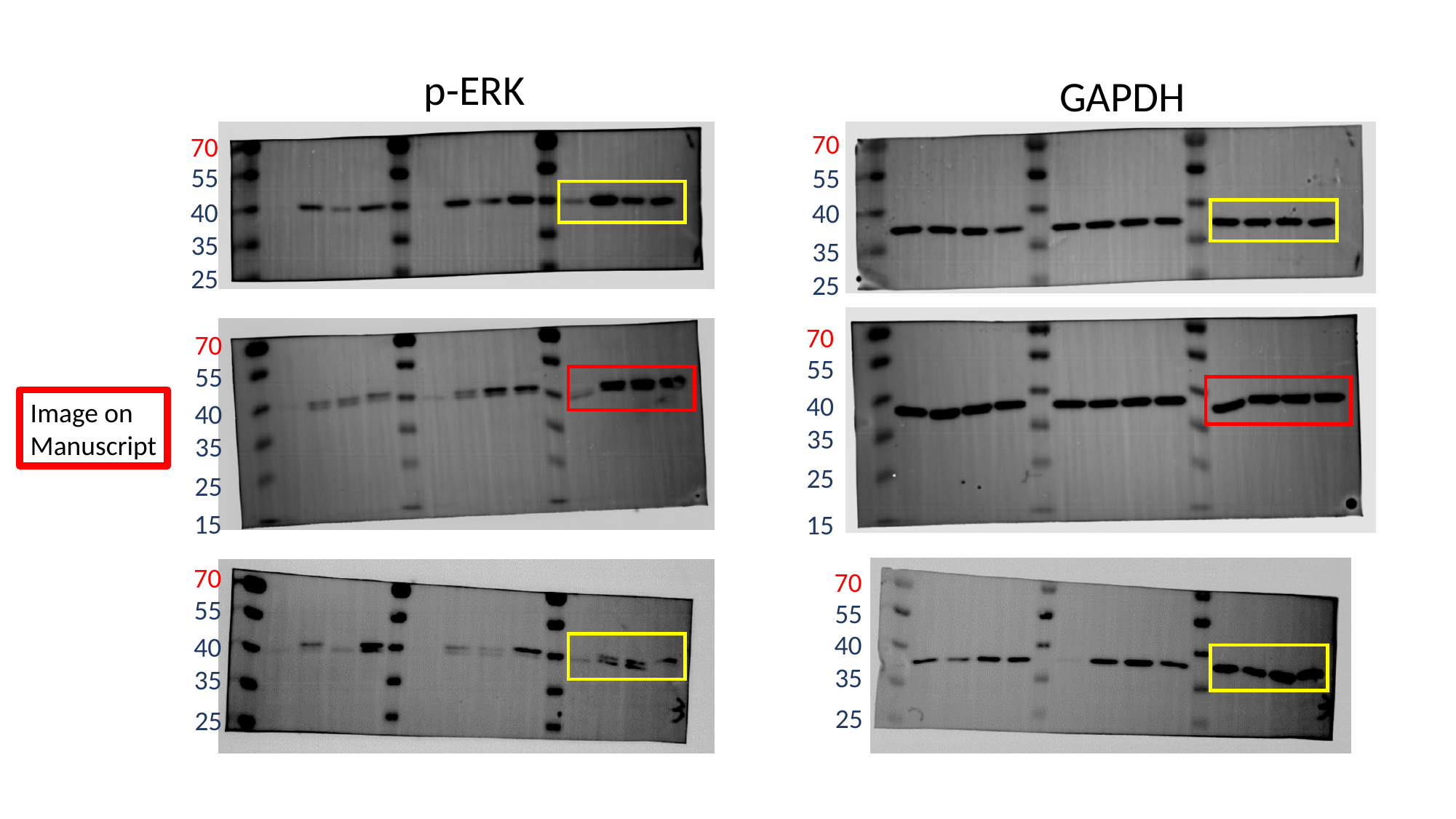

p-ERK
GAPDH
70
70
55
55
40
40
35
35
25
25
70
70
55
55
40
Image on
Manuscript
40
35
35
25
25
15
15
70
70
55
55
40
40
35
35
25
25

## Slide 10
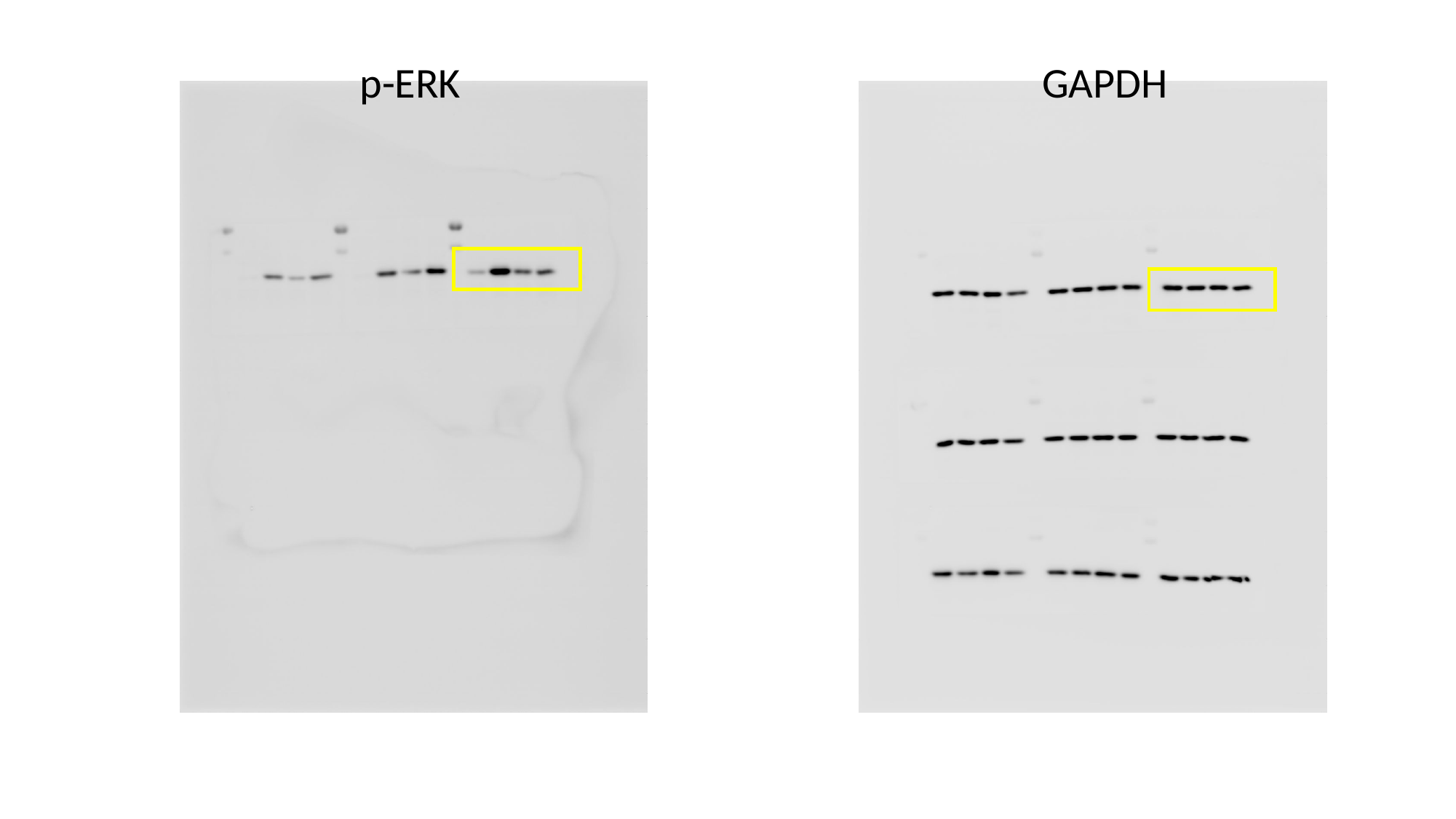

p-ERK
GAPDH

## Slide 11
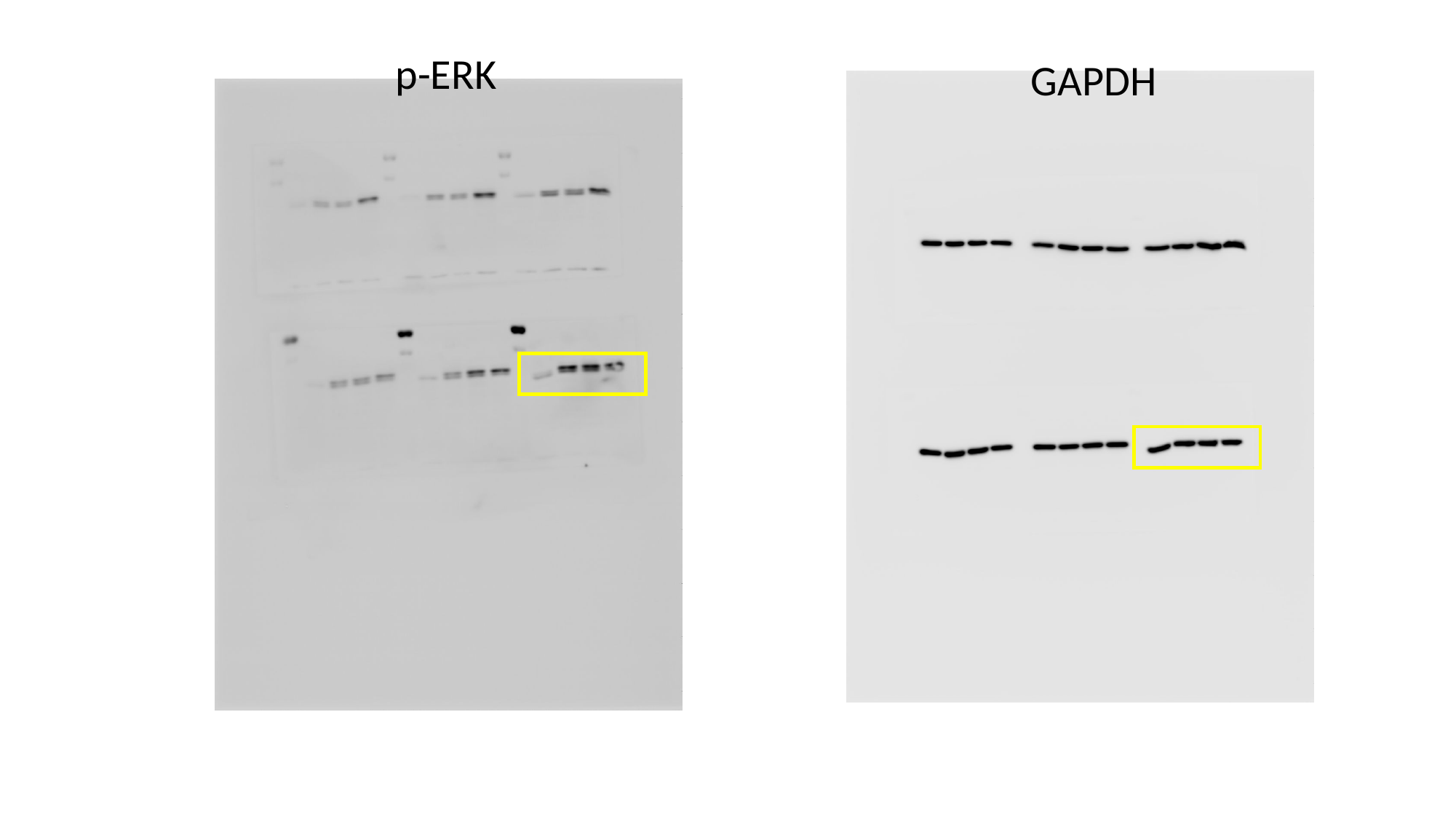

p-ERK
GAPDH

## Slide 12
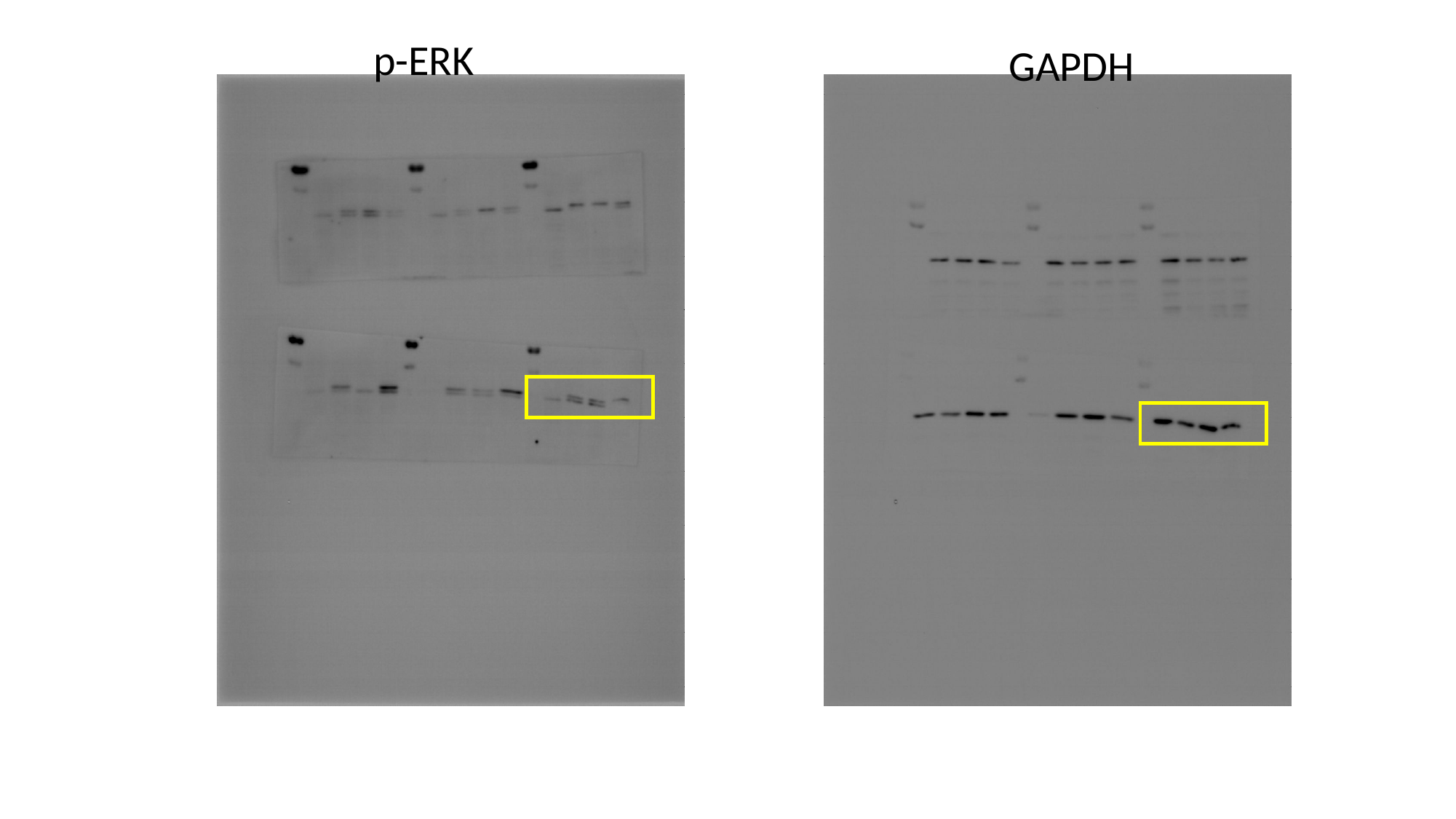

p-ERK
GAPDH

## Slide 13
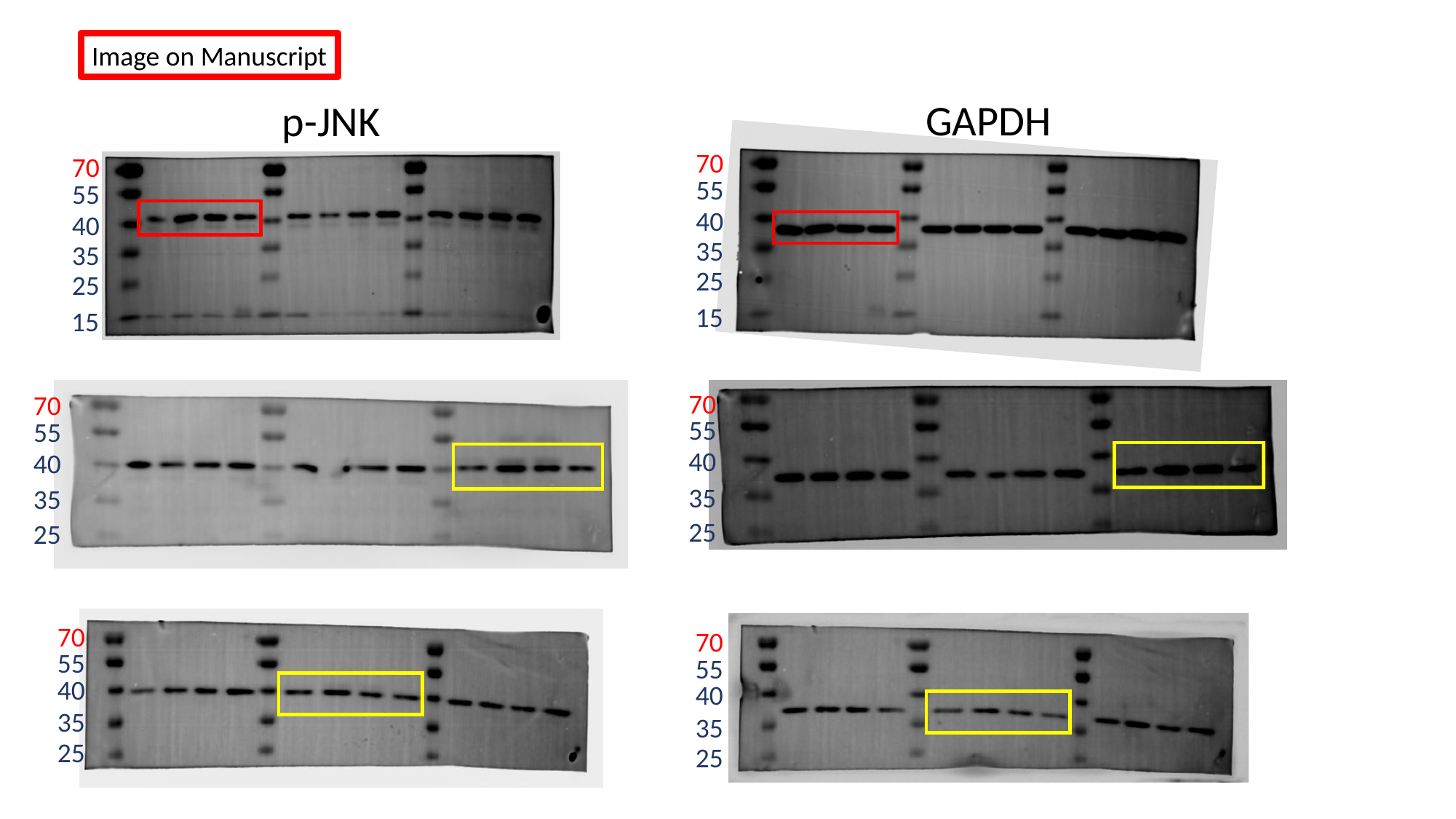

Image on Manuscript
GAPDH
p-JNK
70
70
55
55
40
40
35
35
25
25
15
15
70
70
55
55
40
40
35
35
25
25
70
70
55
55
40
40
35
35
25
25

## Slide 14
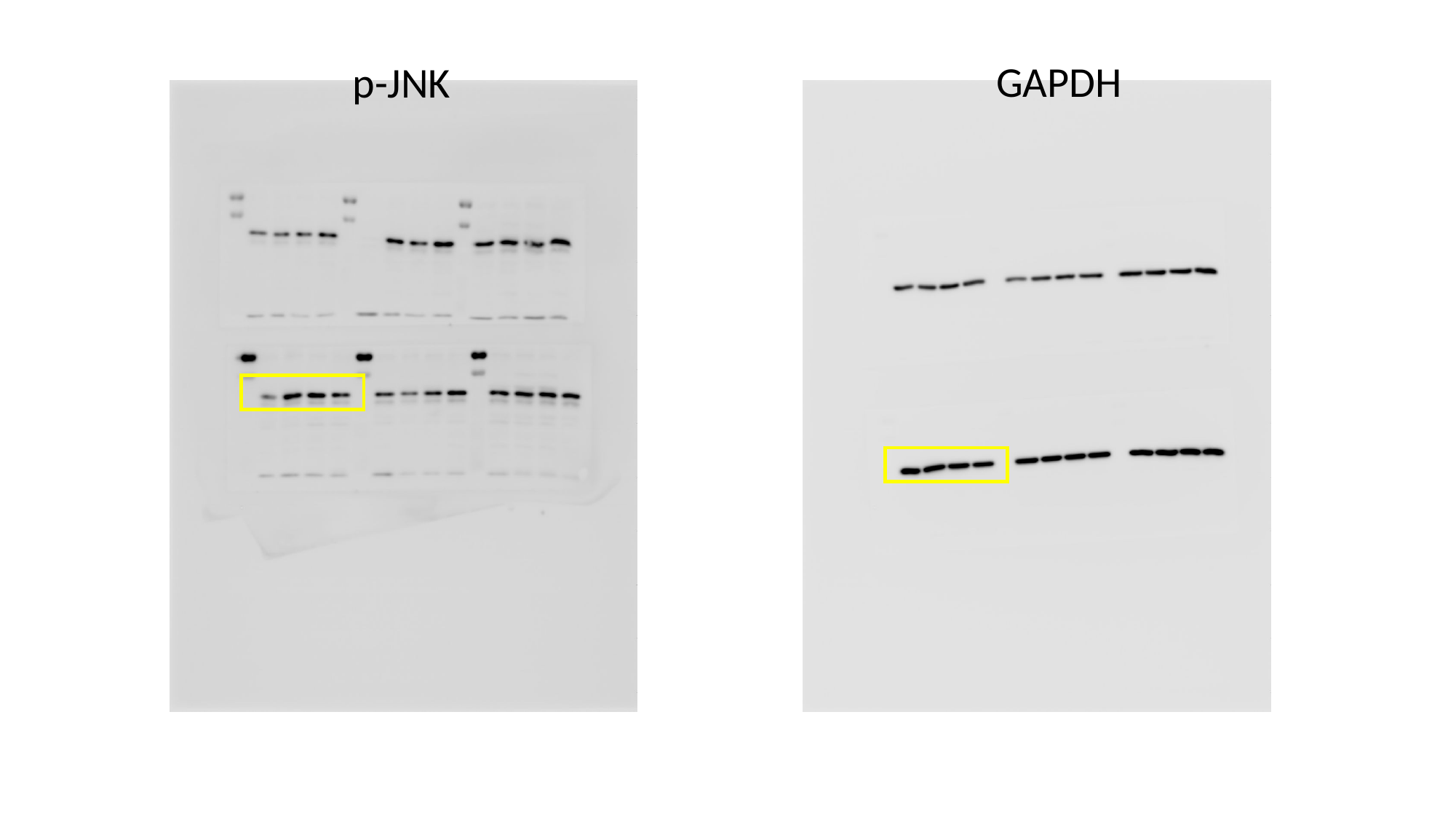

GAPDH
p-JNK

## Slide 15
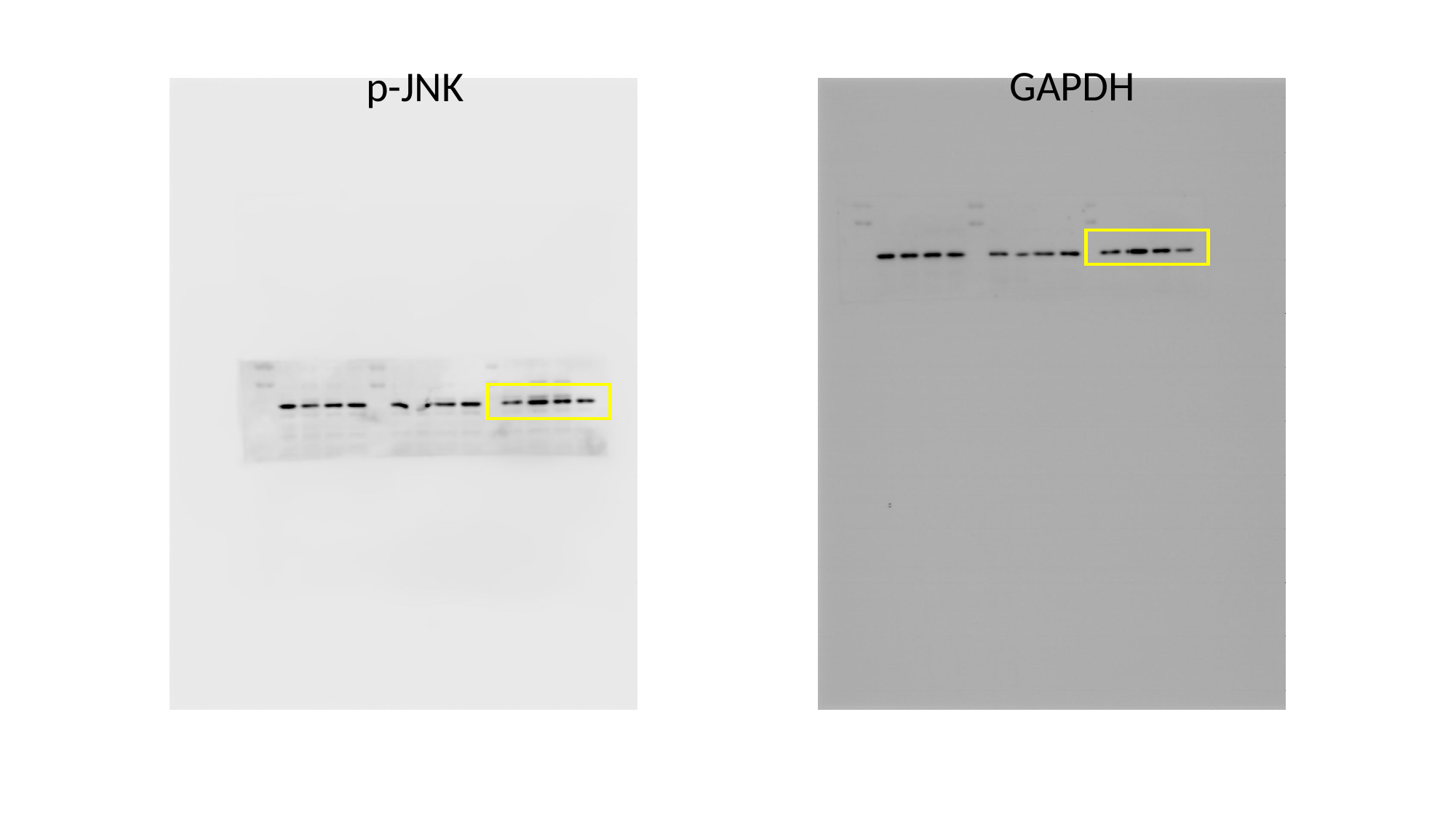

GAPDH
p-JNK

## Slide 16
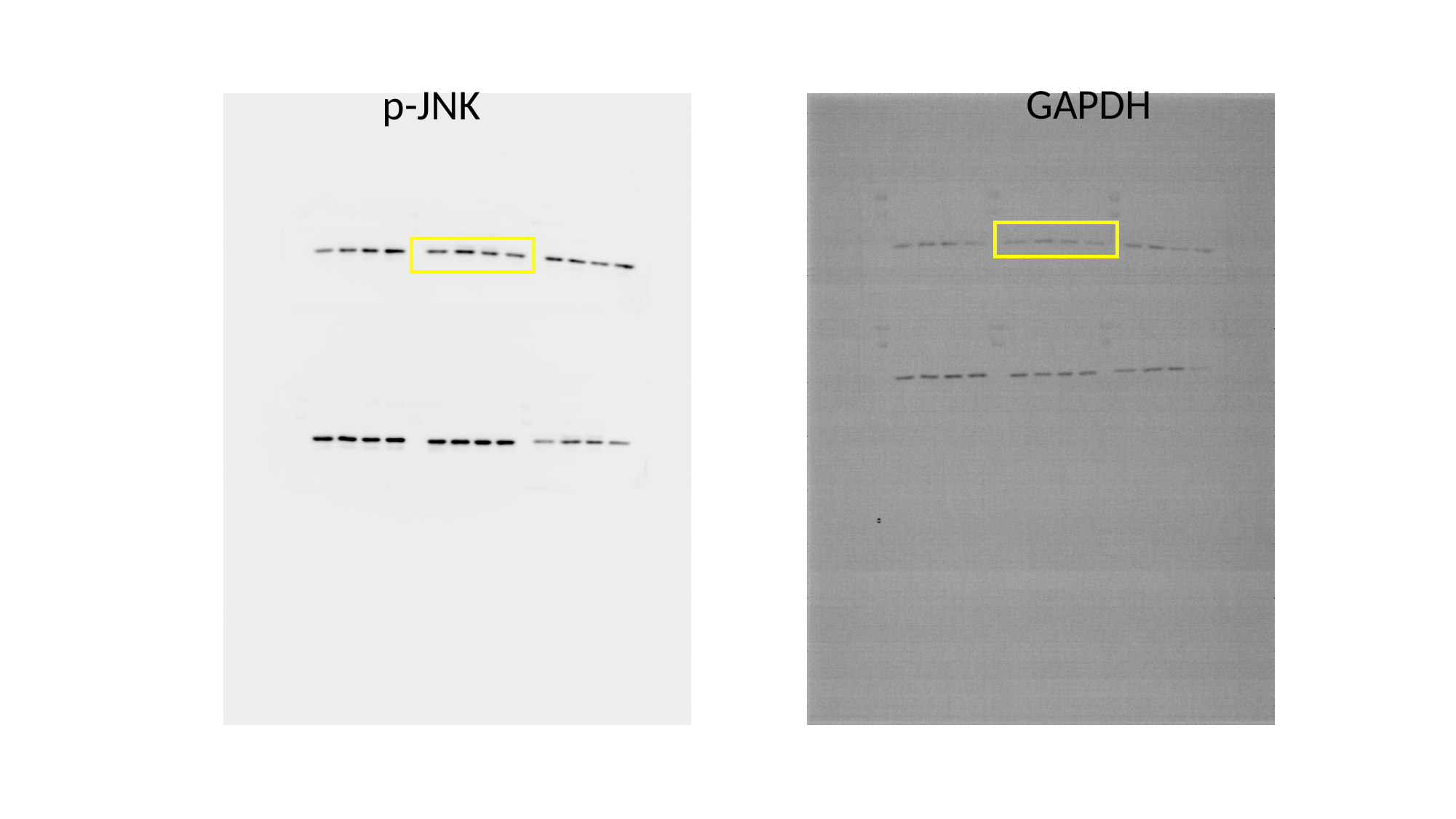

GAPDH
p-JNK

## Slide 17
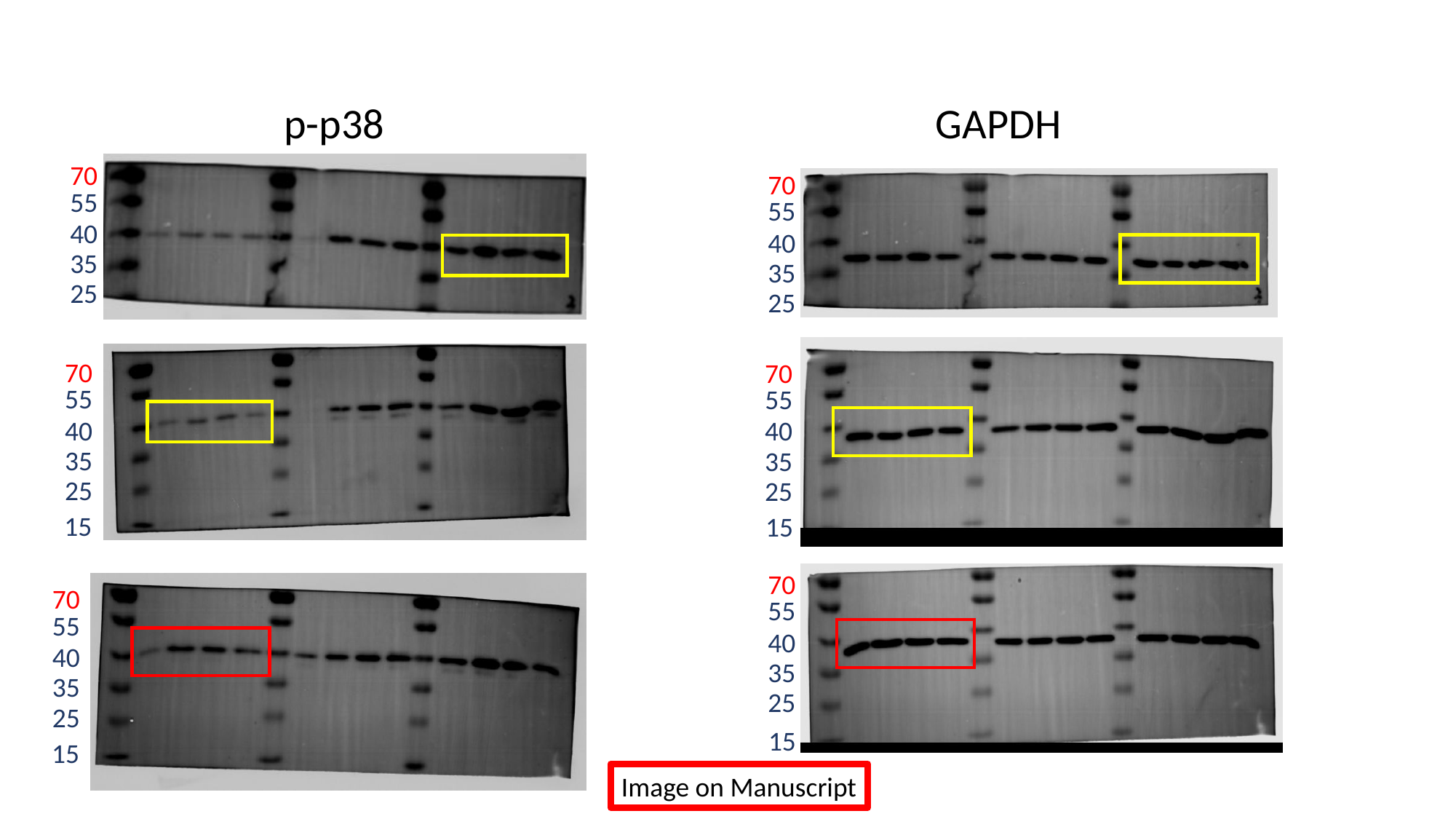

p-p38
GAPDH
70
70
55
55
40
40
35
35
25
25
70
70
55
55
40
40
35
35
25
25
15
15
70
70
55
55
40
40
35
35
25
25
15
15
Image on Manuscript

## Slide 18
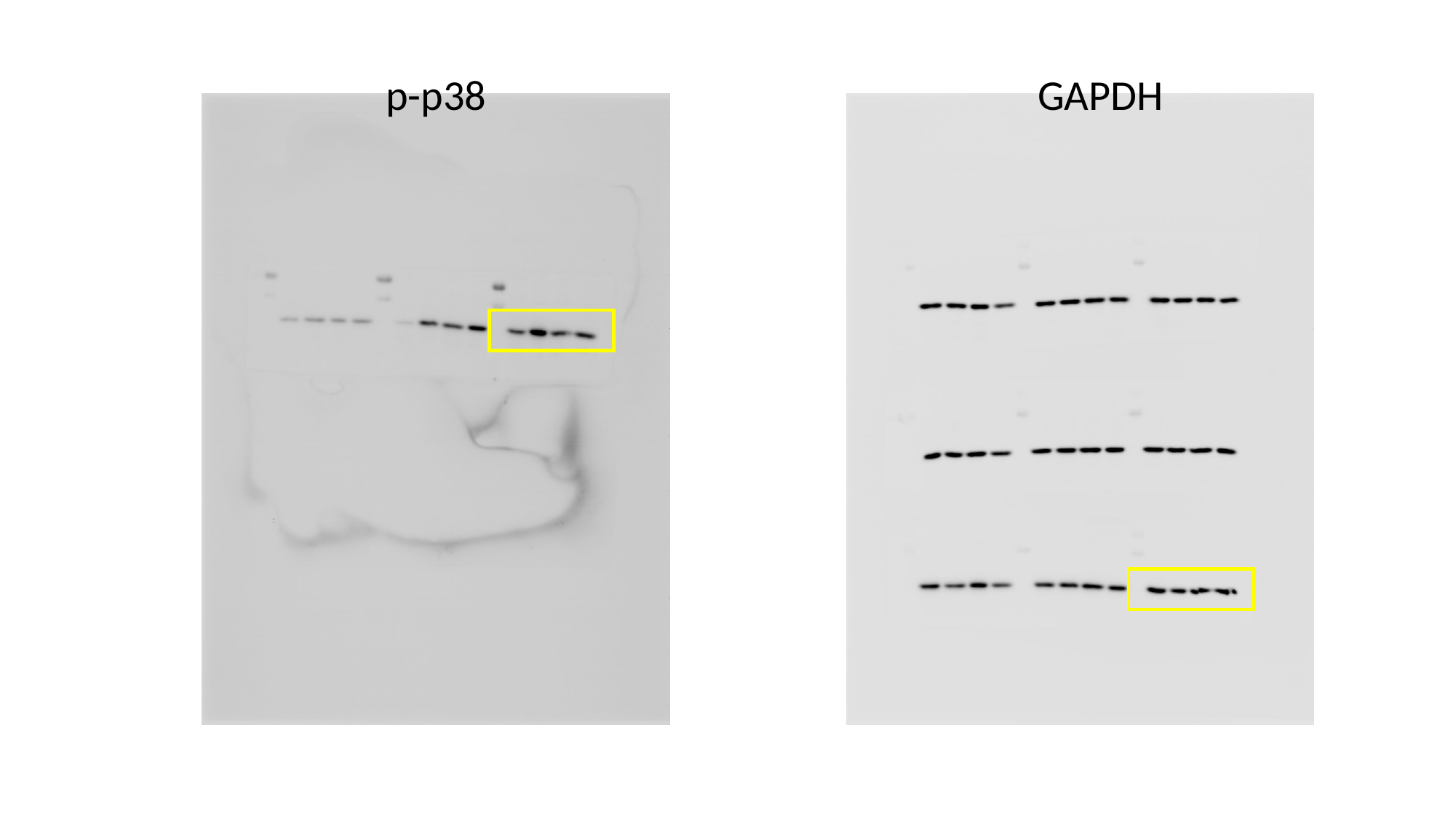

p-p38
GAPDH

## Slide 19
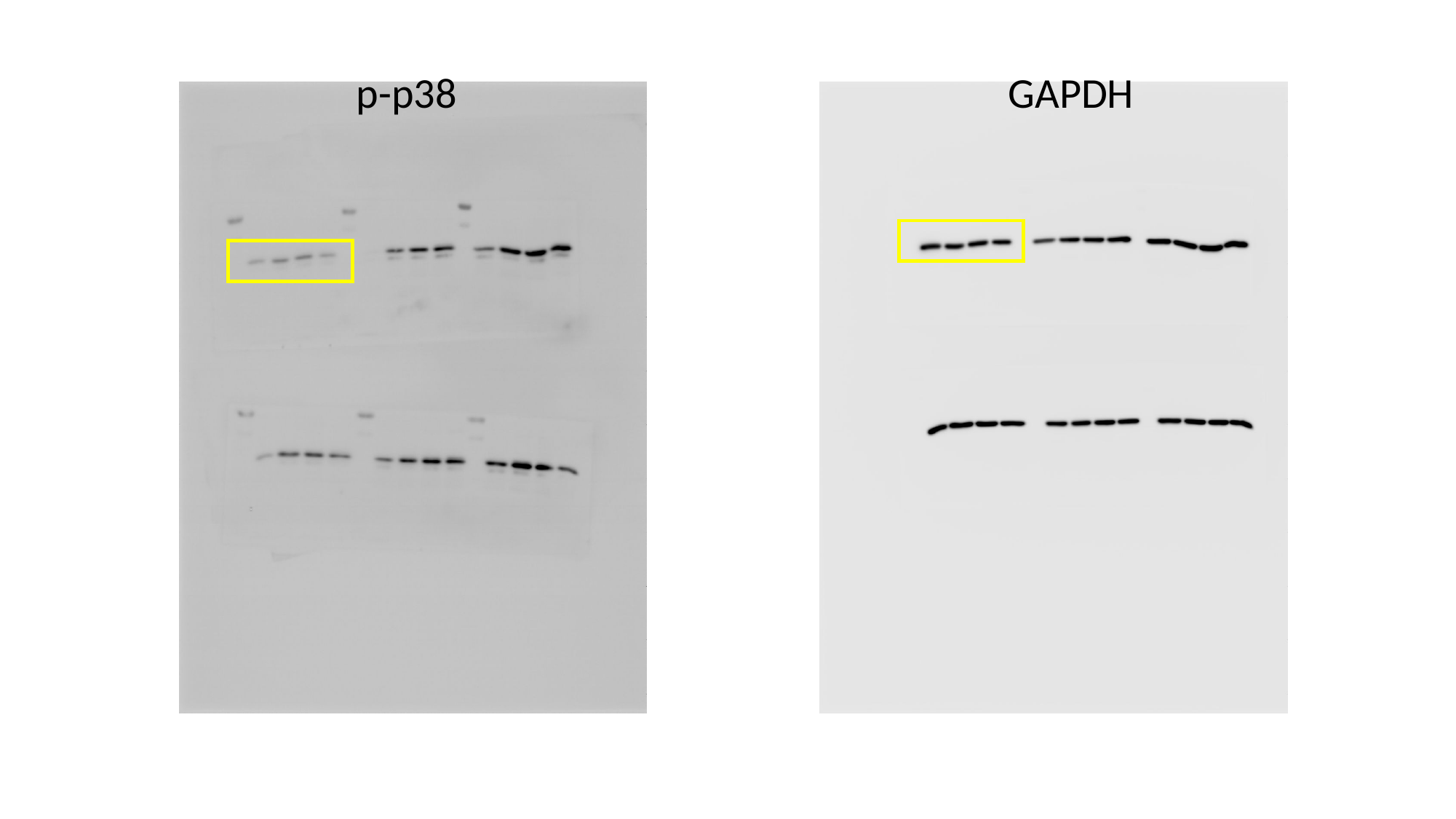

p-p38
GAPDH

## Slide 20
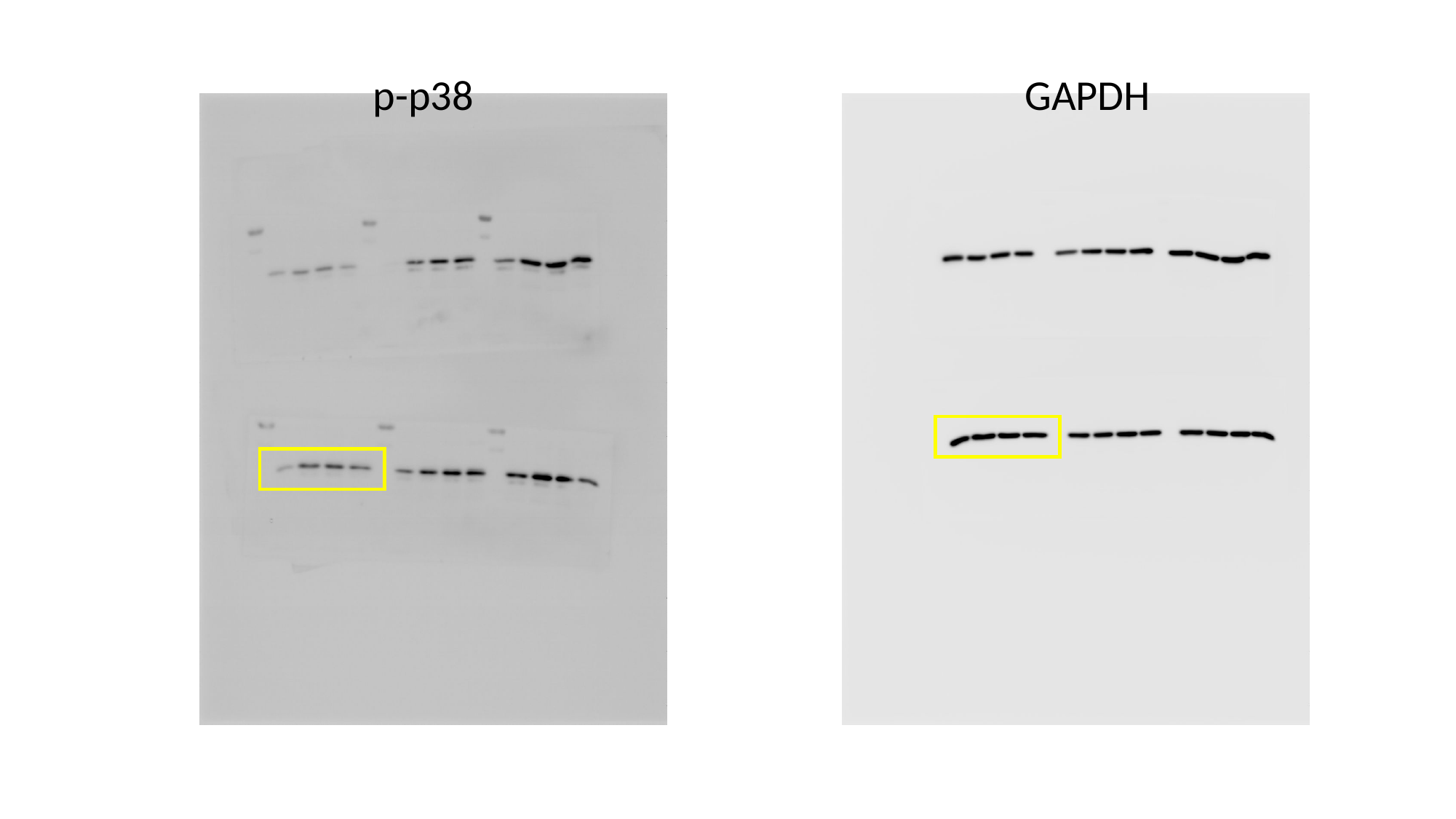

p-p38
GAPDH
